# Towards a sampling design for characterizing habitat-specific benthic biodiversity related to oxygen flux dynamics using Aquatic Eddy Covariance

**DOI:** 10.1371/journal.pone.0211673

**Published:** 2019-02-04

**Authors:** Iván F. Rodil, Karl M. Attard, Joanna Norkko, Ronnie N. Glud, Alf Norkko

**Affiliations:** 1 Tvärminne Zoological Station, University of Helsinki, Hanko, Finland; 2 Baltic Sea Centre, Stockholm University, Stockholm, Sweden; 3 University of Southern Denmark, Odense, Denmark; 4 Technical University for Marine Science and Technology, Tokyo, Japan; University of Waikato, NEW ZEALAND

## Abstract

The Aquatic Eddy Covariance (AEC) technique has emerged as an important method to quantify *in situ* seafloor metabolism over large areas of heterogeneous benthic communities, enabling cross-habitat comparisons of seafloor productivity. However, the lack of a corresponding sampling protocol to perform biodiversity comparisons across habitats is impeding a full assessment of marine ecosystem metabolism. Here, we study a range of coastal benthic habitats, from rocky-bed communities defined by either perennial macroalgae or blue mussel beds to soft-sediment communities comprised of either seagrass, patches of different macrophyte species or bare sand. We estimated that the maximum contribution to the AEC metabolic flux can be found for a seafloor area of approximately 80 m^2^ with a 5 meter upstream distance of the instrument across all the habitats. We conducted a sampling approach to characterize and quantify the dominant features of biodiversity (*i*.*e*., community biomass) within the main seafloor area of maximum metabolic contribution (*i*.*e*., gross primary production and community respiration) measured by the AEC. We documented a high biomass contribution of the macroalgal *Fucus vesiculosus*, the seagrass *Zostera marina* and the macroinvertebrate *Mytilus edulis* to the net ecosystem metabolism of the habitats. We also documented a significant role of the bare sediments for primary productivity compared to vegetated canopies of the soft sediments. The AEC also provided insight into dynamic short-term drivers of productivity such as PAR availability and water flow velocity for the productivity estimate. We regard this study as an important step forward, setting a framework for upcoming research focusing on linking biodiversity metrics and AEC flux measurements across habitats.

## Introduction

Coastal benthic zones are diverse and productive environments capable of sustaining vital ecosystem functions and providing valuable societal services [1]. Primary production and respiration are metabolism metrics commonly used for describing ecosystem functions and community health *e*.*g*., [2–5]. The contribution of different elements of the benthic biodiversity such as richness of species, abundance, biomass or range of habitats to the ecosystem metabolism is key for the functioning of coastal ecosystems *e*.*g*., [2,3,6,7]. For instance, marine macrophytes, including macroalgae and seagrass, can be dominant sources of primary production and respiration in coastal systems *e*.*g*., [7–9]. Microphytobenthos, also a key coastal primary producer, has a crucial role for the overall ecosystem metabolism of unvegetated marine habitats *e*.*g*., [10,11]. Coastal ecosystems also harbour a high diversity of macrobenthic consumers that contribute to the ecosystem metabolism reflected by elevated benthic O_2_ uptake *e*.*g*., [3, 6,12]. However, the majority of the studies on benthic biodiversity and/or ecosystem metabolism have been conducted within the same type of habitat *e*.*g*., [8,9–13], and cross-site information from different habitats with complex emergent structures (*e*.*g*., seagrass, seaweeds, and blue mussels) is largely lacking.

Coastal systems are complex environments characterized by heterogeneous benthic communities [1,3]. As benthic biodiversity changes across communities and habitats, it is essential to understand the role of the habitat-specific components in the variability of the O_2_ fluxes [14]. Lately, the aquatic eddy covariance (AEC) technique has been implemented to quantify rates of primary production and respiration from continuous measurements of vertical O_2_ fluxes within the benthic boundary layer [15]. The AEC enables extended temporal measurements and integrates large areas of the heterogeneous seafloor under the naturally varying characteristics of the dynamic coastal environments, overcoming the limitations of previous methods for measuring benthic metabolism [15–17]. This rapidly developing technique provides great promise in resolving differences in benthic metabolism across contrasting habitats. The AEC has been successfully adapted to quantify O_2_ dynamics in a wide variety of seafloor environments including high latitude rocky embayment [17], coral reefs [18], seagrass meadows [19], and oyster beds [20]. These pioneering studies are transforming the research on ecosystem functioning. A few studies have established some preliminary links between habitat biodiversity and AEC fluxes *e*.*g*., [18,19,21–24]. However, there are no biodiversity surveys specifically developed within the multidirectional context of the seafloor metabolic flux measurements obtained by the AEC technique.

We need to develop simple but efficient methodologies that can relate habitat biodiversity and heterogeneity information to the seafloor area of maximum metabolic contribution measured by the AEC from multiple directions. The size and shape of the seafloor surface area that contributes most of the flux registered (*i*.*e*., 90%) at the AEC measuring point is known as the ‘flux footprint’ [16]. The non-invasive nature of the AEC allows for quantitative footprint flux comparisons between different habitats under true *in situ* conditions [15,16]. However, there is no equivalent sampling protocol developed to comparably quantify the abundance and biomass of the biota across different benthic habitats. Therefore, our goal is to propose a sampling method based on established protocols for routine biodiversity sampling of subtidal benthic communities to reliably characterize the dominant biomass elements of different coastal habitats within the main metabolic area of influence measured by the AEC. As a first step, our study attempts to provide a framework which enables comparisons to be made across benthic habitats before further studies can start constraining spatial and temporal benthic biodiversity and flux dynamics in more detail. Here, we measured the main seafloor metabolic and biodiversity metrics across a range of benthic habitats in a temperate coastal setting of the Baltic Sea archipelago. We determined the multidirectional AEC footprint area with the maximum contribution to the flux signal, and then we superimposed a sampling design to characterize the habitat-specific biodiversity contributing to the seafloor-specific footprint. We define the benthic biodiversity footprint, a term associated to the AEC footprint, as the main benthic biodiversity area of influence that contains the largest metabolic contribution measured by the AEC. Eventually, this information will be pivotal for future research, when comparing and linking benthic biodiversity measures with AEC metrics of productivity and respiration across coastal habitats.

## Materials and methods

### Study sites

**Fig 1:**
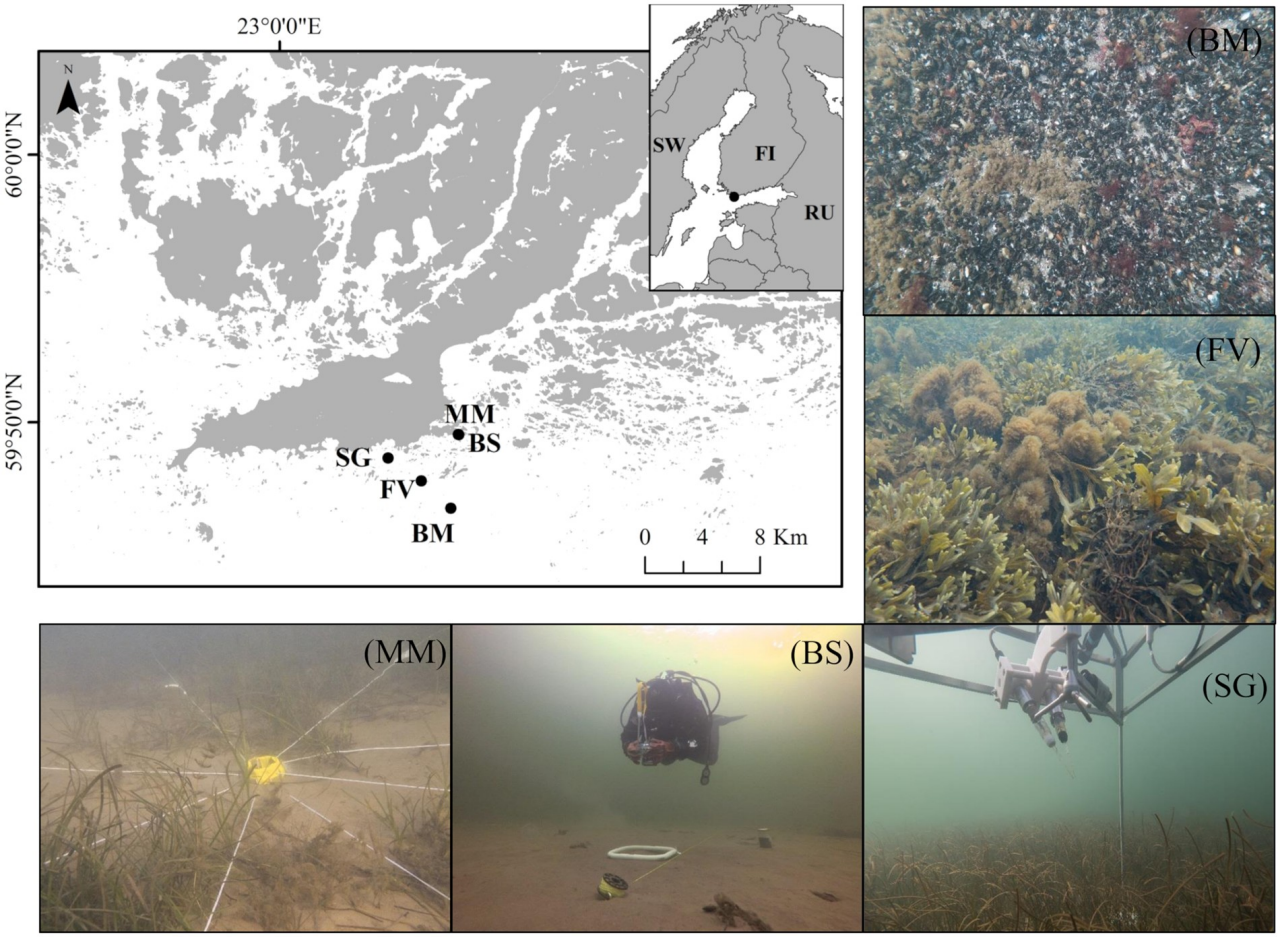
Map of the Baltic Sea archipelago (SW Finland) showing the locations of the habitats. Mixed macrophyte (MM) and bare sand (BS) habitats, exposed seagrass meadow (SG), littoral bladder-wrack belt (FV), and blue mussel reef (BM). A selection of photographs showing the biodiversity sampling area at the MM and BS, the AEC deployed at the SG, and a closer look to the *Fucus vesiculosus* and blue mussels at the BM and FV rocky beds, respectively.

The archipelago coastline presents a complex structure of islands and a mosaic of soft and rocky bottom shores. We selected five shallow (≤ 5 m) habitats of the Baltic Sea archipelago (*i*.*e*., one site per habitat), on the Hanko Peninsula, SW Finland (Fig 1, Table 1). Three habitats were selected as main representatives of the soft-sediment communities: (1) a vegetated site comprised of mixed macrophyte species (henceforth, MM), (2) an adjacent bare sand site (henceforth, BS), and (3) a seagrass meadow (henceforth, SG), mainly comprised by *Zostera marina* Linnaeus, 1753 (Fig 1). We selected two habitats as main representatives of the hard-bottom communities: (4) a bladder-wrack bed (*Fucus vesiculosus* Linnaeus, 1753) site (henceforth, FV), and (5) a blue mussel reef (*Mytilus edulis* Linnaeus, 1758) site (henceforth, BM) (Fig 1). The study was conducted from October to early November 2016.

**Table 1:** Aquatic eddy covariance (AEC) deployment information and environmental characteristics (mean±SE). This table includes information on the AEC footprint area and productivity flux estimates (Gross primary production, GPP, community respiration, R, and net ecosystem metabolism, NEM) at the five habitats. Photosynthetic active radiation (PAR) is calculated as an integration of continuous measurements over 24 h. X_max_ is the integrated area with the maximum flux contribution (see [16]).

AEC info		Bare sand (BS)	Mixed macrophyte (MM)	Seagrass (SG)	Bladder-wrack bed (FV)	Blue mussel reef (BM)
AEC deployment	Date deployment	19.10.16	18.10.16	01.11.16	13.10.16	14.10.16
	Date recovery	22.10.16	22.10.16	07.11.16	17.10.16	17.10.16
	Dataset length (h)	90	90	106	35	70
	Days	3.8	3.8	4.4	1.7	2.9
Environmental conditions	Depth (m)	4	3.5	4	2	5
	Salinity (PSU)	5.6	5.6	5.5	N/A	5.6
	Temperature (°C)	9.4 ± 0.2	9.4 ± 0.2	5.7 ± 0.6	9.3 ± 0.8	10.7 ± 0.1
	PAR (mol m^-2^ d^-1^)	2.7 ± 1.6	3.1 ± 1.3	1.3 ± 0.2	3.1	1.0 ± 0.2
	Day/night time periods (h)	11.3/12.8	11.3/12.8	10.0/14.0	11.8/12.3	11.0/13.0
	Mean flow (cm s^-1^)	2.2 ± 0.1	2.2 ± 0.1	2.7 ± 0.2	2.1 ± 0.1	2.9 ± 0.1
	Min-Max flow (cm s^-1^)	0.3–6.6	0.3–6.6	0.0–11.7	0.9–5.4	0.2–6.2
AEC footprint	Measurement height (cm)	26	26	30	25	26
	Length (m)	42.7	20.4	8.8	36.1	17.6
	Width (m)	1.7	1.7	2.0	1.63	1.7
	Area (m^2^)	57	27.1	13.6	46.4	23.5
	X_max_ (m)	2	0.7	0	1.2	0.59
AEC flux	GPP (mmol m^-2^ d^-1^)	11.9±2.5	8.3±2.1	7.9±1.9	13.6±4.9	2.6±2.9
	R (mmol m^-2^ d^-1^)	5.5±1.4	8.2±1.0	10.3±0.8	2.4±3.1	44.6±3.9
	NEM (mmol m^-2^ d^-1^)	6.5±2.0	0.2±1.6	-2.4±1.4	11.2±6.3	-42.1±3.4
Coordinates		BS	MM	SG	FV	BM
		59°50'29.45"N	59°50'29.73"N	59°49'27.74"N	59°48'40.98"N	59°47'43.12"N
		23°15'1.23"E	23°14'59.24"E	23° 9'52.90"E	23°12'26.46"E	23°14'44.59"E

### Aquatic eddy covariance system, deployment, flux measurements and AEC footprint

Benthic O_2_ fluxes were quantified *in situ* using the AEC technique [15]. Our AEC systems (Fig 1) are very similar to the original design [25], and have been described in detail in previous studies [26,27]. The main hardware consisted of an acoustic Doppler velocimeter (Nortek) mounted vertically in the centre of a tripod frame and 2 Clark-type O_2_ microsensors connected to the velocimeter via submersible picoamplifiers (response time < 0.3 s). A photosynthetic active radiation (PAR) sensor (LI-192, Li-Cor), a dissolved O_2_ optode (U26-001, HOBO), and a saltwater conductivity sensor (U24-002-C, HOBO) located on the frame logged transmitted (seabed) PAR, dissolved O_2_ concentration, temperature and salinity throughout each deployment at 5 min intervals. The AEC was deployed from a small boat, and a lift-bag was used by divers to gently lower the tripod onto the seabed, approximately in a central and representative area of the habitat. Once deployed, the instrument provided an accurate measure of the actual distance between the sensor measurement height and the seabed, and proceeded to log flow velocity and O_2_ microsensor output in continuous sampling mode at 32 Hz. Individual deployments lasted 3–4 days.

**Fig 2:**
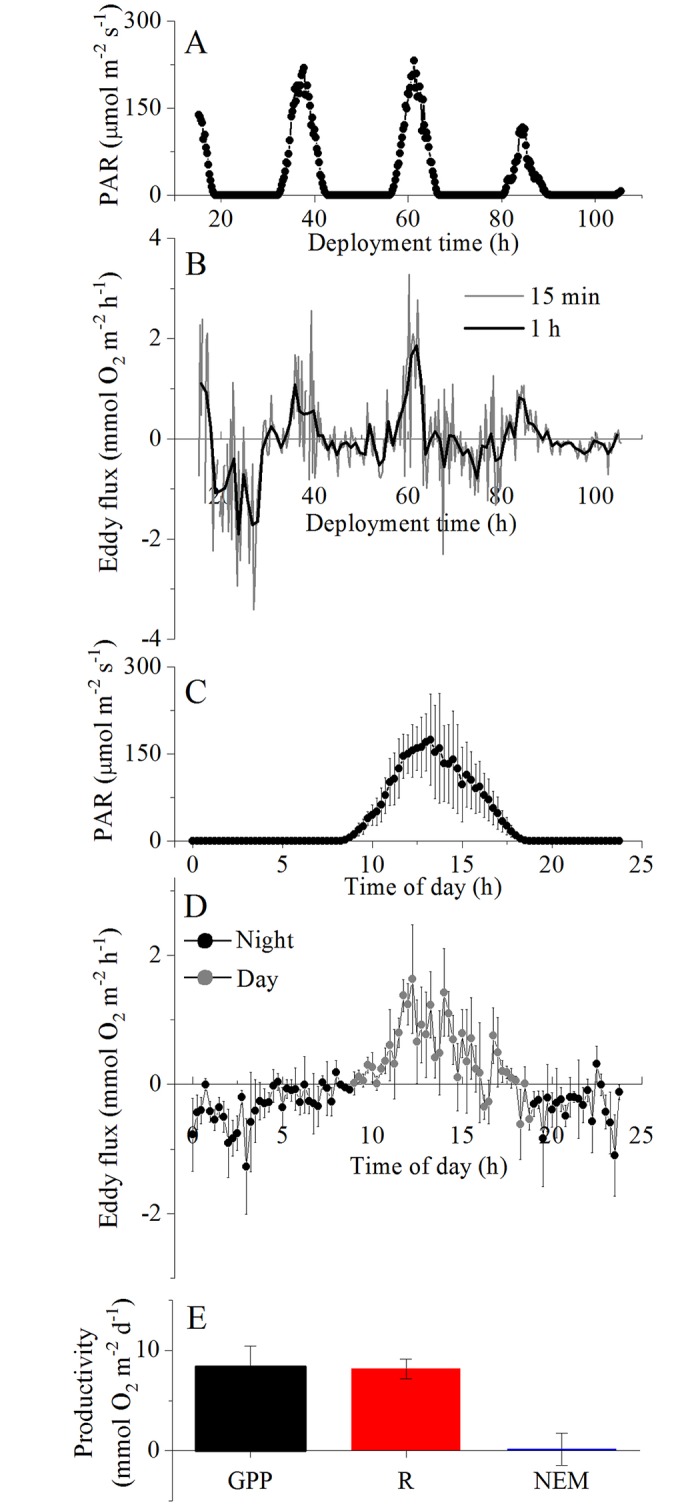
Example of eddy covariance data treatment for calculating daily seafloor productivity rates from the MM site. The collected datasets for PAR (15 min, A) and the eddy fluxes (15 min, B) were bin-averaged by the hour of day to produce a single 24 h time series for PAR (C, mean ± SD, n = 3) and eddy flux (D, mean ± SE, n = 3). The eddy flux time series was separated into day-time and night-time fluxes to compute daily rates of seafloor gross primary productivity (GPP), respiration (R), and net ecosystem metabolism (NEM) (E, in mmol O_2_ m^-2^ d^-1^, see Methods).

Benthic O_2_ fluxes (mmol m^-2^ h^-1^), were extracted from the measured velocity and O_2_ microsensor data streams following well-established protocols [15,27,28]. Fluxes for consecutive 15 min intervals were computed using the open-source software SOHFEA [29]. Multiple days of flux measurements were bin-averaged to produce a single, continuous 24 h time series of fluxes for each site (see Fig 2). The fluxes were aligned with PAR and separated into daytime (PAR > 0.0) and night-time (PAR = 0.0) fluxes (μmol m^-2^ s^-1^). These fluxes were averaged to provide a mean ± SD Flux_light_ and Flux_dark_, and the PAR time series was used to determine the number of daytime hours (h_light_). Daily gross primary productivity was calculated as GPP = (|Flux_light_ |+|Flux_dark_ |) x h_light_. Daily respiration rates were calculated as R = |Flux_dark_ | x 24. The net ecosystem metabolism (NEM), representing the daily balance between GPP and R, was computed (mmol m^-2^ d^-1^). Positive NEM indicates a surplus of organic C and O_2_ production (net autotrophy), whereas negative NEM indicates net heterotrophic communities.

For each habitat, the size and shape of the seafloor surface area included in the AEC flux measurements were examined from measurements of seafloor hydraulic roughness (z_0_) and sensor measurement height (Table 1), as described by [16]. The length, width, and region of maximum contribution (X_max_) within the AEC footprint were calculated for each site using the equations provided by [16]. The length and width of the flux footprint were used to estimate the surface area by assuming that the footprint was elliptical in shape (Table 1).

### Flow direction effects on seafloor productivity estimates

**Fig 3:**
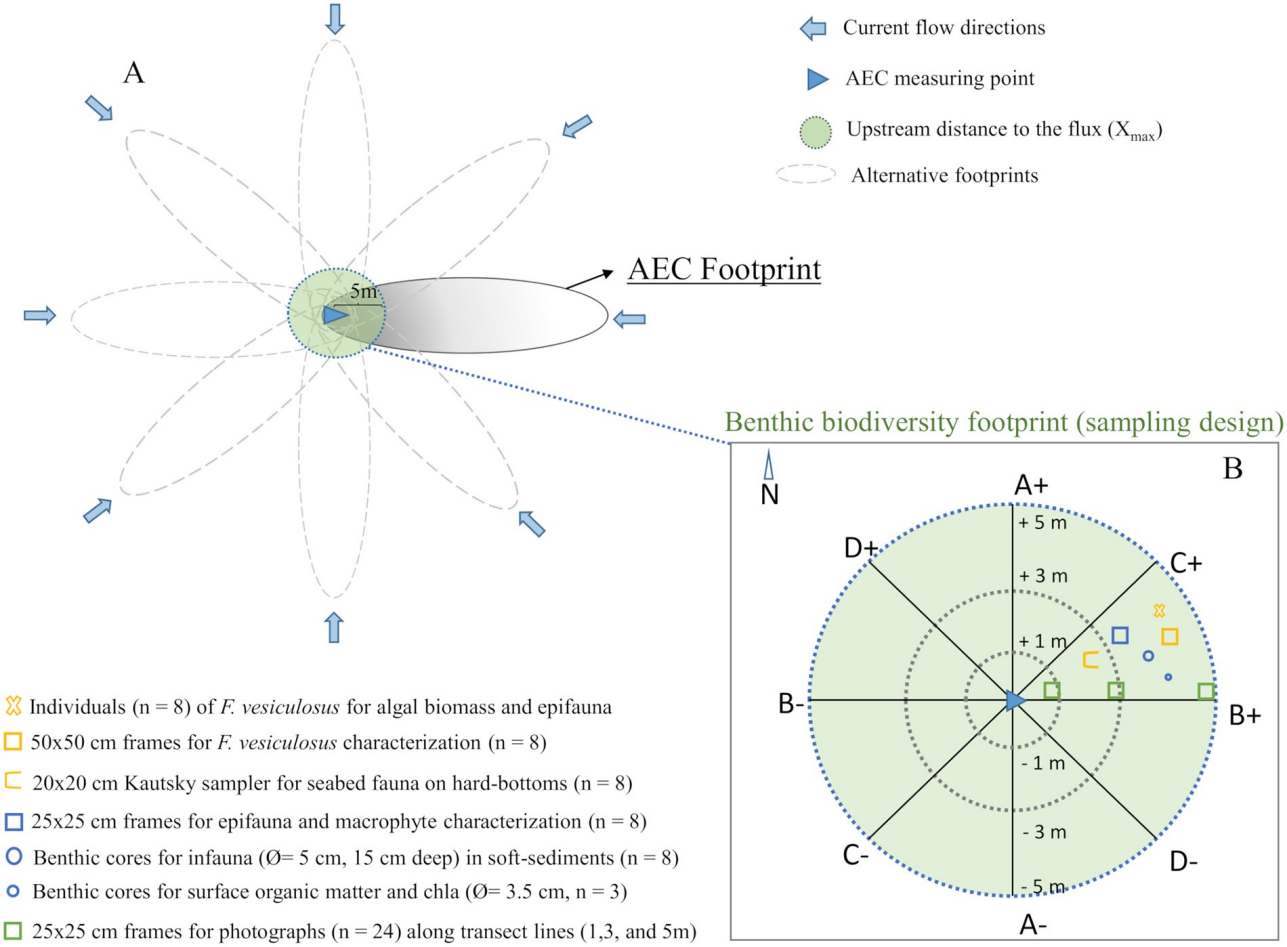
Schematic illustration of the AEC footprint and biodiversity sampling area. (A) Diagram from above showing the AEC footprint areas determined by different flow directions. The length, width and total area of the AEC footprints for all the habitats are shown in Table 1. The circular biodiversity footprint (80 m^2^, r = 5 m) was superimposed to the AEC footprint ensuring that the maximum flux contribution measured in all the habitats (X_max_ = 5 m) was covered. The grey scale shows a theoretical gradient of the benthic contribution to the flux registered within the footprint area (darker grey implies higher contribution closer to the device). (B) Illustration of the biodiversity footprint area displaying 8 equal wedge-sections for community characterization. Quantitative random samples (n = 8, one replicate sample per each of the 8 wedge-sections) of the main benthic organisms (v macrofauna, macrophytes and macroalgae) were collected (see Methods).

We examined the effects of the water flow direction on the AEC estimates of seafloor productivity (*i*.*e*., GPP, R, and NEM), and the corresponding changes in the location of the flux footprint (see Fig 3A). Combined with specific diversity surveys of the footprint areas, analysis of directional O_2_ flux data would provide valuable information about any spatial distribution of productivity on the seafloor, its variability within-habitat, and its drivers. To do this, the software package SOHFEA [29] was used to transform the AEC orthogonal coordinate system (instrument coordinates) to Earth coordinates (ENU) by correcting for the instrument heading. This allowed aligning the AEC coordinate system to the seafloor specific diversity measurements. SOHFEA then generated a corresponding mean flow direction and flow velocity magnitude value for each 15 min O_2_ flux. Directional data were grouped into 8 direction sectors, each representing 45° increments. Data processing and windrose plots were performed in OriginPro 8.5 (OriginLab).

### Defining the benthic community area of contribution: The benthic footprint

**Fig 4:**
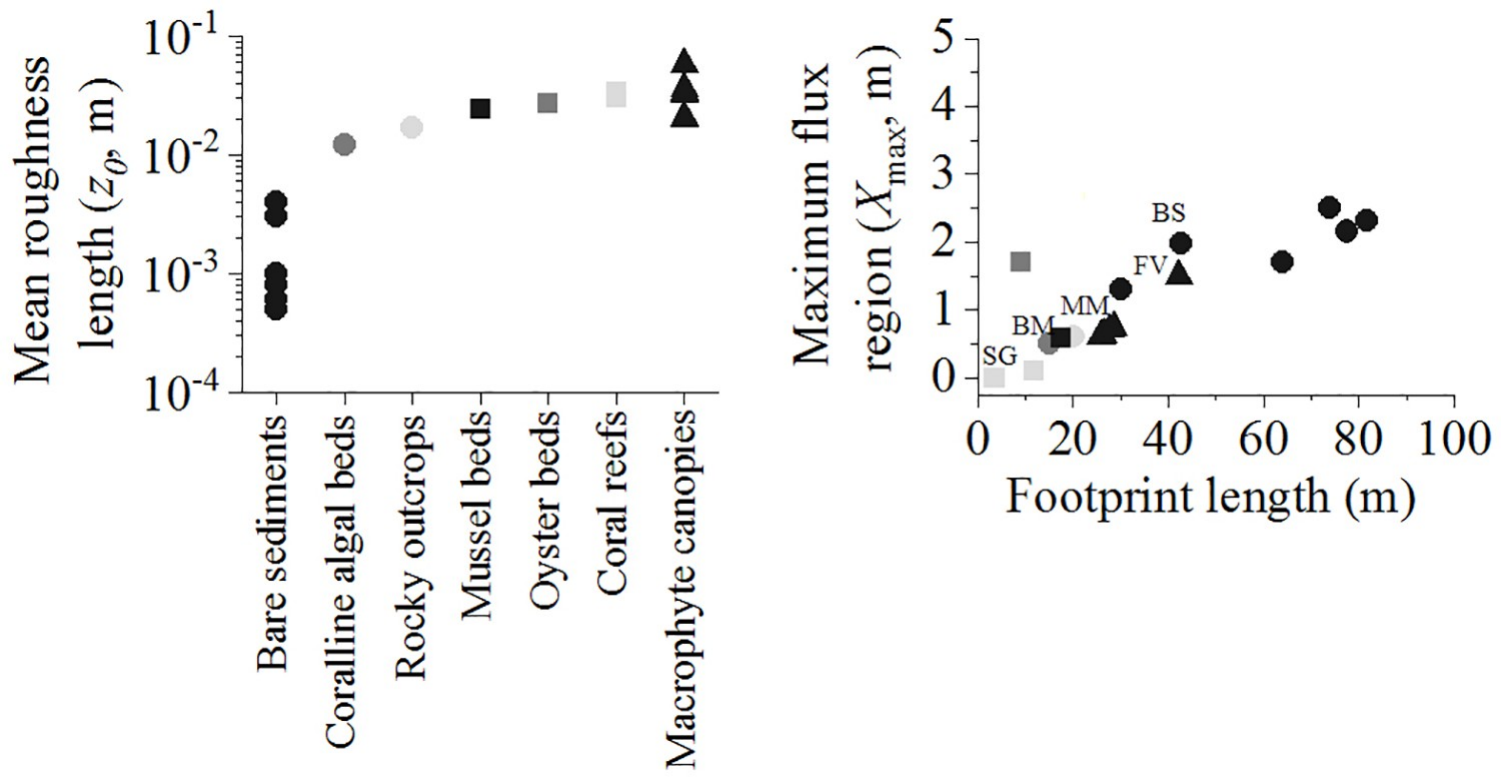
Estimated characteristics of the AEC footprint. Mean roughness length (z_0_) and estimated footprint length versus region of maximum flux contribution (X_max_) for various coastal benthic habitats (Bare sediments: [27,29,31,32, this study]; Coralline algal bed: [27]; Rocky outcrop: [31]; Mussel bed: [this study]; Oyster bed: [20]; Coral reef: [33]; Vegetated canopy: [30,34, this study]). The estimated footprint values (length vs X_max_) for the study sites were allocated (*i*.*e*., BS: bare sand; MM: mixed macrophyte; SG: seagrass meadow; FV: bladder-wrack belt; BM: blue mussel).

Characteristics of the AEC footprint (*i*.*e*., size and shape) can be estimated with knowledge of site-dependent factors such as water depth, seafloor roughness length scale (z_0_), and sensor measurement height [16]. Conservative tracer tracking simulations [16] provided an estimate for the length and width of an ellipse that contributes to 90% of the flux signal, and identified the region of maximum flux contribution (X_max_) within this area. The benthic conditions across various coastal habitats around the world, including our study habitats, show a range of seafloor roughness length scales (*z*_0_, range from 0.0005 to 0.04 m) that will affect the average of the AEC flux measurements (Fig 4A). For typical coastal seafloor settings, the estimated length of the footprint ranges from < 10 m for habitats with rough benthic surfaces (*e*.*g*., oyster beds, [20] or vegetated canopies, [30]) to > 80 m for habitats with smoother surfaces such as bare sediments in unidirectional flows [31] (Fig 4B). The AEC flux signal is not distributed evenly within the footprint area, but most of the flux signal originates from a smaller region (X_max_) typically located < 5 m upstream from the instrument (Fig 4B). In our study, we estimated that a 5 m upstream distance will capture 50 to 80% of the total flux-contributing seafloor area, whether we consider the simple bare sand site or the more complex sites. Therefore, a circular-shaped benthic footprint (Ø = 10 m), covering multiple flow directions (Fig 3A) was superimposed on the typically elliptical-shaped AEC footprint (Fig 3B) to characterize quantitatively the main biodiversity elements (in terms of biomass) within the AEC footprint of maximum contribution. We defined the benthic biodiversity footprint as the minimum benthic area of influence that contained the largest AEC footprint contribution.

### Sampling the benthic footprint: Quantifying benthic communities across habitats

We developed a simple but realistic sampling protocol based on well-established protocols for routine biodiversity sampling of subtidal benthic communities to methodically conduct comparable biodiversity characterization of the main community drivers (*e*.*g*., macrophytes and macrofauna) across different benthic habitats. We used a sampling device composed of an octagonal metallic ring as the centre point and eight diving reels attached at each side of the ring (Fig 1). From each reel, a 5-m long transect line was extended and four equal wedge-sections (+A, +B, +C, +D) plus four mirrored sections (-A, -B, -C, -D) were used to delineate the sampling area (Fig 3B). The +A guideline was always orientated to the North to align the AEC and biodiversity measurements. Thus, we obtained a circular sampling area of approximately 80 m^2^ divided into eight equal wedge-sections (Fig 3B). Each transect line was pre-labelled at 1, 3 and 5 m from the central ring for diving orientation and photographic characterization of the seafloor (Fig 3B). Following this configuration, photographs of 25 cm x 25 cm quadrats were taken at 1, 3 and 5 meters along the guidelines (total of 24 pictures per habitat, Fig 3B) to estimate the cover of the main structural components (*i*.*e*., seagrass, macroalgae, blue mussel, and bare sediment) in each habitat. Multiple issues arise within the framework of the fundamental question of where, how, and with what frequency to take samples. The answers depend upon the kind of information explored in the habitat and the substrate types to be sampled, although resource availability usually determines these boundaries. All the sampling was conducted by divers directly after the AEC deployments.

#### Soft-sediment habitats

At the vegetated sites (MM and SG), the shoot density, canopy height and biomass of all the plants were measured by harvesting all the vegetation within a quadrat frame (25 cm x 25 cm) randomly placed in each wedge-section (n = 8, one random replicate per wedge-section) (Fig 3B). We gently uprooted and bagged all shoots within the quadrat into net-bags. In the lab, all plant material was measured (length, cm), counted (shoots m^-2^), sorted into above- and belowground parts, and dried to constant dry weight (60 °C, 48 h). All the associated epifauna was sorted, counted (individuals m^-2^), identified, and biomass as dry weight (dwt, g m^-2^) was calculated (see section 2.6). At both vegetated and bare sites (Fig 1), sediment samples were obtained for quantification of macroinfauna and for sediment characteristics. One sediment core (Ø = 5 cm, 15 cm deep) for macroinfauna was randomly taken (n = 8) in each wedge-section (Fig 3B). Sediment was sieved (0.5 mm), animals were sorted, identified, counted (ind m^-2^), and biomass (dwt, g m^-2^) was calculated (section 2.6). Sediment surface (1 cm) samples were randomly taken within the sampling area using syringes (Ø = 3.5 cm) for organic matter (%, n = 3) and microphytobenthic pigment (*i*.*e*., chlorophyll *a* and phaeopigments, g m^-2^, n = 3) analyses.

#### Hard-bottom habitats

Benthic samples were collected (n = 8, one random replicate per wedge-section) at the BM reef using a modified Kautsky sampler [35] for hard bottoms (*i*.*e*., a 20 cm x 20 cm square with three metal sides and a sampling bag attached to the fourth side) (Fig 3B). A spoon was used to scrape the communities from the rock into the bag. In the lab, the mussels were distributed evenly on a water-filled tray sectioned in eight sectors. Four sectors were randomly chosen, and mussels within the sector counted and abundance calculated (ind m^-2^). Due to the large abundance of mussels, all the individuals per sample were categorised into size classes, using mesh sizes of 1, 2, 4 and 9.5 mm [36]. Then, approximately 100 from every size class were randomly selected and the length (maximum anterior-posterior axis) was measured with a Vernier calliper to an accuracy of 0.1 mm. All the invertebrate species associated to the mussels were counted, identified, and the biomass was calculated (see section 2.6).

The number and height of all the *F*. *vesiculosus* individuals (n = 8) enclosed in randomly selected PVC-frames (50 cm x 50 cm, one replicate per wedge-section, Fig 3B) were non-destructively counted (ind m^-2^) and measured (height in cm) *in situ* at the FV site. Eight complete *F*. *vesiculosus* mature individuals were randomly taken (one per wedge-section) cutting off the holdfast and enclosed in 0.5 mm mesh-bags (Fig 3B). In the lab, all *F*. *vesiculosus* individuals were dried to constant weight at 60°C (accuracy of 0.1 g). The average individual dry weight (in grams, n = 8) and the average abundance of *F*. *vesiculosus* (ind m^-2^, in 50 cm x 50 cm) were used to calculate the average *F*. *vesiculosus* biomass (dwt, g m^-2^) of the mature individuals. We sorted all the invertebrates associated to the individual samples of *F*. *vesiculosus* (*i*.*e*., epifauna). The epifauna was counted (ind m^-2^), identified, and the biomass was calculated (section 2.6). Random replicates per wedge-section (n = 8) were collected for samples of the macroinvertebrates directly associated to the rock (*i*.*e*., seabed fauna) using a Kautsky sampler, as performed on the BM reef. In the lab, we counted (ind m^-2^), identified and calculated the biomass of all the seabed macrofauna (section 2.6).

### Macroinvertebrate biomass calculation

From all the habitats, all shell-less invertebrates (*i*.*e*., polychaetes, crustaceans and others) were blot wet weighed (accuracy of 0.0001 g). The length (maximum anterior-posterior axis) of molluscs and gastropods was measured with a Vernier calliper to an accuracy of 0.01 mm. Finally, biometric conversion factors for invertebrates of the Baltic Sea [37,38] were used to obtain biomass information for all the species (dry weight, g m^-2^). Dry weight biomass for the mussels was estimated) as: *Meat weight = shell length*^*2*.*307*^ *x 10*^*−4*.*744*^ [36].

### Coverage of the main benthic structural components

We applied a supervised image classification technique to map all the pictures (n = 24; Fig 1 and S1 Fig), performing a maximum likelihood classification on a set of raster bands (ArcGIS 10.1 geoprocessing tool). The benthic structural components were selected, and the area covered (%) was classified according to their colour (pixel counts) using the spectral signature previously defined in a training set (ArcMap). For instance, at the BS site the microphytobenthos layer can be distinguished from bare sediment using the processed pictures and their different colour density criteria, the former being dark to light shades of green and the latter brown or black.

### Benthic community analysis

All the invertebrates were identified to the lowest possible taxon, usually species (S1 File). The total macrofauna abundance (ind m^-2^), biomass (g m^-2^) and number of species were calculated per habitat. We tested whether the main benthic community indicators (*i*.*e*., abundance and biomass, log (x+1) transformed) and the species assemblages (4^th^-root transformed abundance) differed across habitats (*i*.*e*., SG, MM, BS, FV, and BM) using a permutational analysis of variance (PERMANOVA, Bray Curtis similarity matrix, 4999 unrestricted permutations) [39]. The abundance and biomass (S1 File) of the main macroinvertebrate groups (*i*.*e*., crustaceans, molluscs, gastropods, and polychaetes) were also estimated. Similarly, we tested all the sedimentary (*i*.*e*., organic matter, chlorophyll *a*, phaeopigments) and macrophyte (*i*.*e*., length, density and biomass) biotic metrics (log (x+1) transformed). All the analyses were carried out in PRIMER v.6 and PERMANOVA+ [40,41].

## Results

### AEC footprint, water flow and productivity estimates

**Fig 5:**
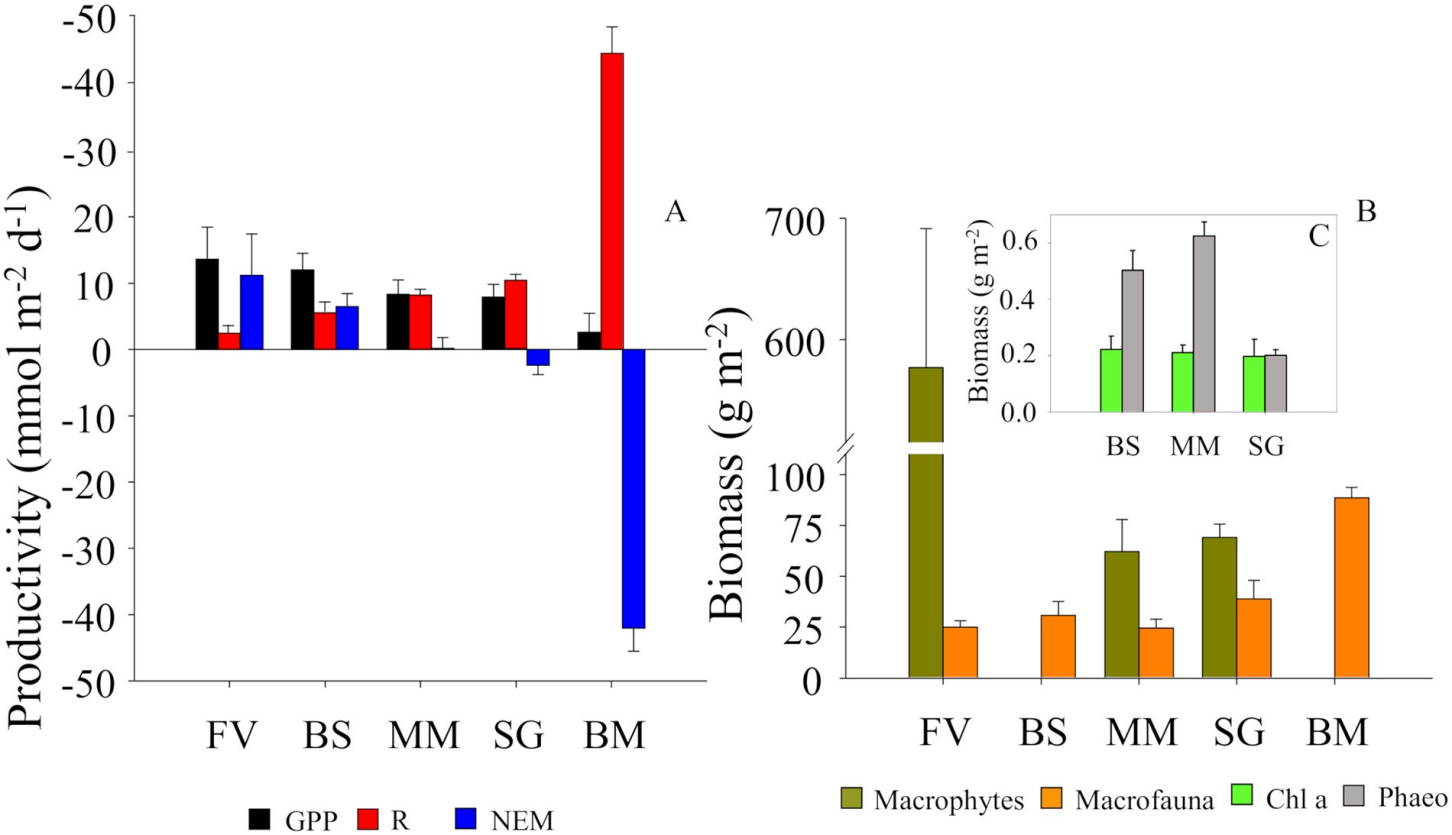
Mean (+SE) ecosystem metabolism and benthic biodiversity biomass. (A) Ecosystem metabolism (GPP: gross primary production, R: community respiration, NEM: net ecosystem metabolism), (B) benthic biomass (dry weight) of macrophytes (*Z*. *marina* and *Fucus vesiculosus*) and macrofauna (total average), and (C) benthic biomass (dry weight) of chlorophyll a (Chl a) and phaeopigments (Phaeo) from all the habitats (FV: bladder-wrack bed, BS: bare sand, MM: mixed macrophyte, SG: seagrass meadow, BM: blue mussel reef). Different letters represent significant differences (p < 0.05) for the main metrics.

The characteristics of the footprint, calculated for a water depth of < 5 m and a measuring height of 25–30 cm, were habitat dependent (Table 1). The length of the footprint ranged between 8.8 m (SG) and 42.7 (BS) m, while the width ranged between 1.6 (FV) and 2.0 (SG) m (Table 1). As a result of this distribution, the footprint area was relatively large in all the habitats, ranging between 13.6 (SG) and 57 (BS) m^2^. However, the maximum contribution to the flux (X_max_) at the measuring point was found for a spot at the seafloor surface located ≤ 2 m upstream of the instrument, ranging from 0 (SG) to 2 (BS) m (Table 1). The AEC flux data provided variable rates of benthic productivity depending on the habitat. Thus, the BS site was a net autotrophic habitat (6.5±2.0, mmol O_2_ m^-2^ d^-1^), while the MM site was a metabolically balanced habitat (*i*.*e*., NEM ≈ zero; 0.2±1.6, mmol O_2_ m^-2^ d^-1^) at the time of the study (Table 1, Fig 5A). The SG site was a net heterotrophic habitat (*i*.*e*., -2.4±1.4, mmol O_2_ m^-2^ d^-1^). The FV site showed the largest O_2_ production (NEM was almost twice as high as BS, *i*.*e*., 11.2±6.3 mmol O_2_ m^-2^ d^-1^), whereas the BM site showed the largest net O_2_ uptake compared to the other habitats (NEM was 17 times more negative than in the SG site, -42.1±3.4 mmol O_2_ m^-2^ d^-1^) (Table 1, Fig 5A).

**Fig 6:**
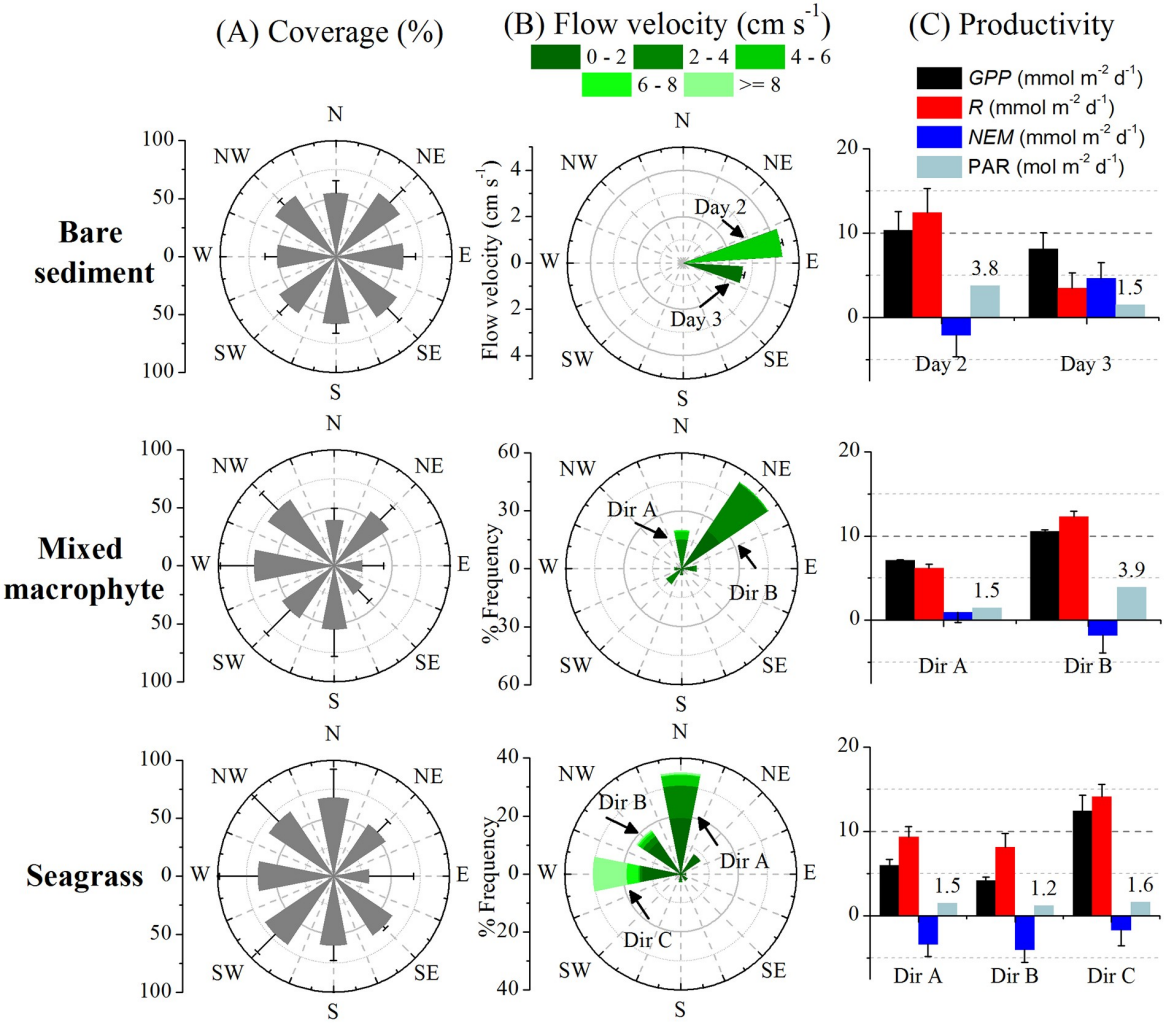
Windrose plots (mean ± SD) for the soft-sediment sites. (A) Coverage (ArcMap, see section 2.7.) of benthic microalgae (*i*.*e*., chlorophyll a), macrophytes, and seagrass, (B) directional flow velocity distribution, and (C) daily seafloor productivity rates for different days or different directions (daily integrated PAR values are indicated).

**Fig 7:**
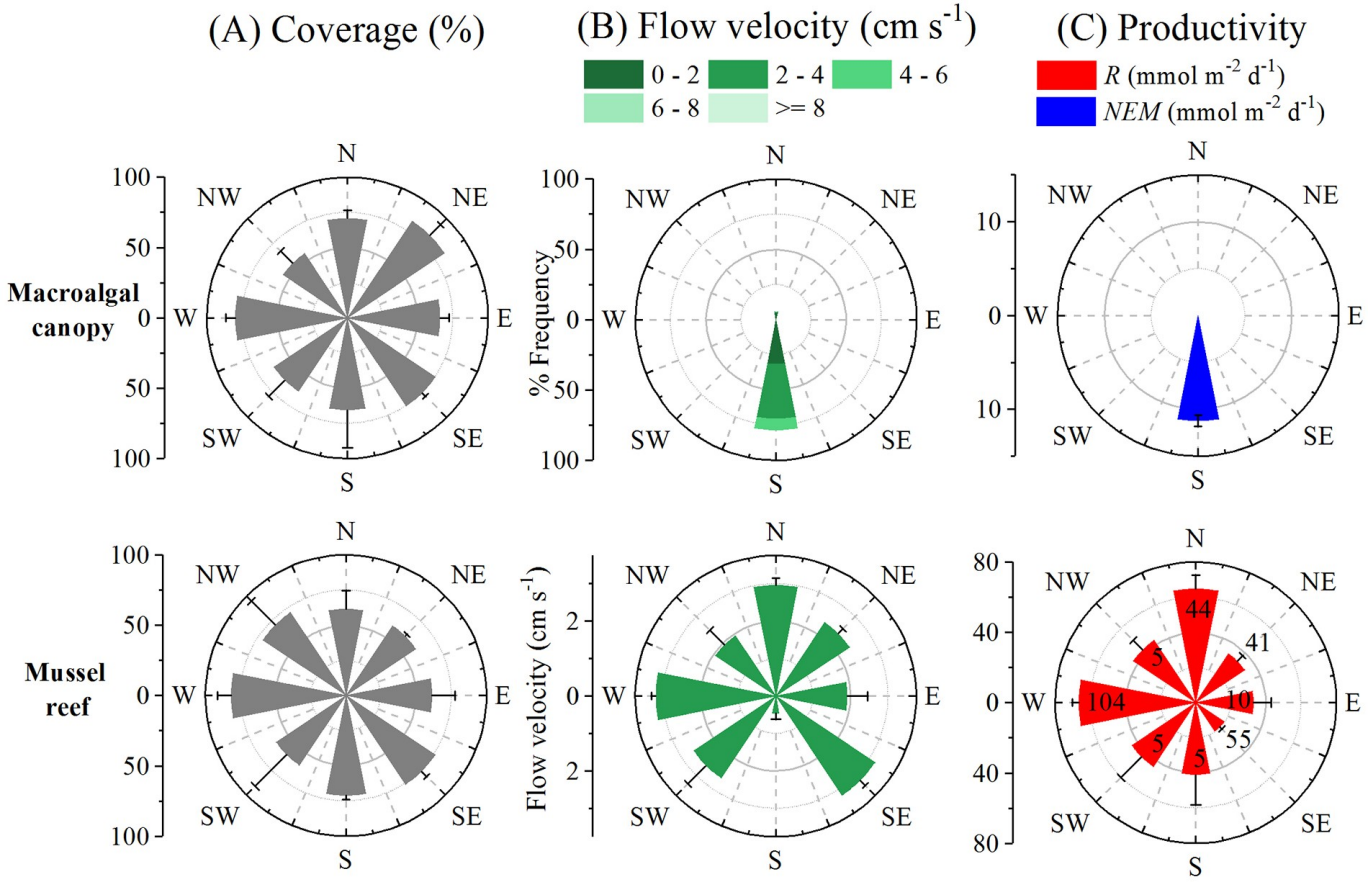
Windrose plots (mean ± SD) for rocky substrates. (A) Coverage (ArcMap, see section 2.7.) of macroalgae and mussels, (B) directional flow velocity distribution, and (C) corresponding seafloor productivity rates, indicating the number of 15 min fluxes.

Directional polar plots show the water flow direction as a function of velocity and frequency and how it relates to the habitat community composition. The BS site showed a relatively uniform cover of microphytobenthos (Fig 6A) and a unidirectional flow for most of the dataset (Fig 6B). The mean flow velocity (Fig 6B) and the PAR were 2-fold higher in day 2 than day 3 (Fig 6C). GPP and R were also higher in day 2 than day 3, and NEM was negative in day 2 versus positive in day 3 (Fig 6C). *Z*. *marina* was the most abundant plant species in the MM site (S1 File, S1 Fig), and its coverage distribution (24 ± 18 and 69 ± 19%) was variable (Fig 6A). There were two predominant flow directions, Dir A and Dir B. PAR, GPP and R were 2-fold higher in Dir B than in Dir A (Fig 6C). NEM was positive in Dir A and negative in Dir B (Fig 6C). The SG site showed a more homogenous coverage (~ 60% in all directions) of *Z*. *marina* compared to the MM site (Fig 6A) within the three main flow directions (A, B, C) (Fig 6A and 6B). There were similar rates of GPP, R, and NEM for Dir A and B under similar PAR (Fig 6C). Dir C showed a similar PAR, but higher flow velocities (33% of the time ≥ 8 cm s^-1^) compared to other directions (Fig 6B and 6C). GPP and R were higher in Dir C than in other directions (Fig 6C), but NEM was similar and negative in all three directions (Fig 6C). *F*. *vesiculosus* clearly dominated the FV site (Fig 7A, S1 Fig) and the unidirectional flow (Fig 7B) showed a positive NEM (Fig 7C). No comparisons between days and sectors are possible since we only have one complete day of data for this site. The BM site was homogenously covered in all the sampled sections by *M*. *edulis*, ranging from 60 ± 2 to 82 ± 10% (Fig 7A). The mean flow velocity for the direction sectors ranged from 0.5 ± 0.4 to 3.2 ± 1.5 cm s^-1^ (Fig 7B). There was no light response in the eddy fluxes (*i*.*e*., no measurable primary productivity), thus the O_2_ fluxes represent R only. The mean R for the direction sectors ranged from 19 ± 2 (n = 55) to 67 ± 4 mmol O_2_ m^-2^ d^-1^ (n = 104) (Fig 7C).

### Habitat biodiversity characterization: The role of benthic consumers and producers

There were significant differences in the biodiversity characteristics of the dominant benthic consumers (see Fig 5B, S1 File). The lowest number of macroinvertebrate species was found at the BS site (6), while the rest of the sites presented a similar number of species (10–13) (Table 2, S1 File). The abundance (Pseudo-F_4,35_ = 61.1; p < 0.001) and biomass (Pseudo-F_4,35_ = 4.1; p < 0.01) was significantly different across habitats (Fig 5B, Table 2, S1 File). The highest and the lowest biomass were related to the BM and FV sites, respectively (Fig 5B, Table 2). The biomass of the mussels was significantly different (i.e. pairwise test: FV = MM = BS = SG < BM; p < 0.01) compared to the other habitats (Fig 5B, Table 2).

**Table 2:** Mean (±SE) abundance, biomass and the number of species observed at the five habitats. All the macrofauna was divided between those species associated to the macrovegetation or macroalgae (*i*.*e*., epifauna), and those species directly related to the seafloor substrate (*i*.*e*., seabed fauna), either buried in the sediment or attached to the rocky bed. Abundance (individuals m^-2^), biomass (dry weight, g m^-2^).

	Sites				
	BS	MM	SG	FV	BM
Epifauna	Bare sand	Mixed macrophyte	Seagrass	Bladder-wrack bed	Blue mussel reef
Abundance	**-**	638.9±299	855±105	628±184	**-**
Dry weight	**-**	1.4±0.6	2.8±0.5	7.9±2.6	**-**
Species	**-**	6	9	9	**-**
Seabed fauna					
Abundance	1489±292	5569±633	7920±1422	2581±636	48984±3291
Dry weight	30.6±6.9	24.6±4.3	36.2±8.8	16.93±3.3	88.4±5.2
Species	6	5	10	10	10
Total					
Abundance	1489±292	6207±609	8774±1502	3209±605	48809±3285
Dry weight	30.6±6.9	25.9±4.4	38.9±9.0	24.9±3.3	88.3±5.2
Species	6	10	13	12	10

There were also significant differences in the characteristics of the dominant benthic producers (see Fig 5B, S1 File). Thus, there were more aquatic plant species (F_1,14_ = 19.7, SG < MM, t = 4.4; p = 0.01) at the MM site than at the SG site (S1 File), but the biomass (*i*.*e*., above and below ground, and total) was not significantly different (Fig 5B, S1 File). However, the macrophyte biomass at MM (t = 6.83) and SG (t = 7.97) was significantly (p < 0.001) lower than the *F*. *vesiculosus* biomass (577±115 g m^-2^) at FV site (Pseudo-F_2,21_ = 38.5; p < 0.001) (Fig 5A).

The sedimentary chlorophyll *a* (a surrogate of the microphytobenthos layer) did not vary significantly (F_2,6_ = 0.55) between sites (Fig 5C). However, the degradation product of the algal pigments (*i*.*e*., phaeopigments) varied significantly (F_2,6_ = 41.8; p < 0.001) (SG < MM = BS; p < 0.01) (Fig 5C). The organic matter (%) varied (F_2,6_ = 1.54; p = 0.288) from 0.63 ± 0.09 (MM), 0.84 ± 0.14 (SG) to 0.64 ± 0.03 (BS).

## Discussion

Coastal systems are connected by highly heterogeneous and productive benthic habitats. AEC measurements integrate over a large and heterogeneous seafloor surface area, and allow comparisons to be made between specific sites with contrasting structural biodiversity elements. However, the lack of a standardized sampling protocol to perform a realistic characterization of the dominant biodiversity features when using AEC measurements is impeding a comprehensive assessment of marine ecosystem metabolism across coastal habitats. To our knowledge, this is the first time that benthic biodiversity surveys across habitats have been performed exclusively upon the multidirectional AEC flux footprint characteristics (*i*.*e*., length, width, and area of the footprint and maximum contribution to the flux, X_max_). The AEC technique improves the estimates of whole-system benthic metabolism incorporating different structural biodiversity components at an unprecedented level [15,16]. Whereas it is often unpractical to survey the benthic biodiversity on the scale of the entire AEC footprint, by focusing on the area closest to the instrument it is possible to obtain significant information about the biodiversity components that contribute the most to the AEC flux signal. In this study, we defined a seafloor area of approximately 80 m^2^ with a 5 meter upstream distance as the largest biodiversity area of influence that contains the largest AEC footprint contribution measured (*i*.*e*., 50–80%, see section 2.4.). At the bare sand site, the selected upstream distance captured the lowest AEC footprint contribution, *i*.*e*., approximately 50% of the total flux-contributing seafloor area. However, bare sand sites are homogeneous habitats with less variable sedimentary characteristics, benthic biodiversity and environmental conditions compared to a heterogeneous landscape *e*.*g*., [42]. Therefore, we are confident that the biodiversity components captured in the bare sand site, following our sampling protocol, are representative of the main benthic biodiversity contribution to the AEC flux signal in this homogenous habitat.

We have measured five major shallow habitats from the Baltic Sea coast with very specific biodiversity features. We determined the biomass of the habitat-specific benthic organisms to understand the role of the dominant benthic components in the system, and to discern the broad effects of biodiversity on the AEC measurements, specifically when sampling seafloor habitats with contrasting structural components (*e*.*g*., seagrass meadows or blue mussel reefs). However, there are other key coastal habitats supported by different and also important benthic biodiversity elements (*e*.*g*., oyster reefs or corals) whose idiosyncrasies must be considered when establishing potential links between biodiversity contributors and ecosystem metabolism. While our protocol presents support for its specific use in quickly estimating community biomass and species composition during the sampling of the main benthic habitats of the Baltic Sea, the procedure possesses inherent versatility for implementation in a wide variety of benthic habitats with other structural biodiversity elements and with different seafloor roughness lengths. The scope of this study was not to draw direct relationships between benthic biodiversity elements and seafloor O_2_ eddy fluxes, but to provide a realistic sampling design to quantify dominant features of biodiversity within the seafloor area of maximum AEC metabolic contribution across a range of representative habitats.

### Benthic biodiversity metrics and productivity estimates across-habitats

Habitats with different properties of structural biodiversity (*i*.*e*., macroinvertebrates, macrophytes and microphytobenthos) also functioned very differently. In general, the net ecosystem metabolism of the study habitats followed a productivity gradient from net autotrophic to net heterotrophic related to the main structural biodiversity components. The bladder-wrack (FV) and bare sand (BS) sites were net autotrophic (*i*.*e*., positive NEM), the mixed macrophyte (MM) site was at metabolic balance (*i*.*e*., NEM ≈ zero), and both the seagrass (SG) meadow and the blue mussel (BM) reef were net heterotrophic (*i*.*e*., negative NEM). There were no significant amounts of epiphytes, ephemeral algae or detritus accumulations in any of the study sites (3–6% cover calculated from pictures and ArcMap). The ephemeral species basically responsible for high biomass in this region occur in spring an early summer [43], and the maximum occurrence of mobile detritus accumulations coincides in summer (see [44]). Therefore, the presence of macrophytes (*i*.*e*., *F*. *vesiculosus*, *Z*. *marina* and other angiosperms) suggests an overall high contribution of the macrovegetation biomass to the productivity of the vegetated habitats. Seafloor communities made of canopy-forming macroalgae such as *Fucus* spp. are regarded as autotrophic entities [9,30,45–47]. Thus, the bladder-wrack community showed the highest average GPP and the lowest average R compared to the rest of the study habitats, and the NEM rendered a net autotrophic metabolism. It has been suggested that a low bladder-wrack respiration during night-time could be related to a gas storage effect within gas spaces of *F*. *vesiculosus* [30]. The presence of aquatic angiosperms, mainly *Z*. *marina*, is known to influence the productivity estimates, increasing the metabolism of the seagrass meadows [8,23,24,48]. The differences in the net ecosystem metabolism between the SG and the MM sites could be related to the different shoot densities between sites, thus seagrass shoot density in the SG site was almost three times higher than in the MM site. When shoot density is very high, although the upper parts of the leaves may be light-saturated, the understory can show high respiratory losses because of the shelf-shading effect [8] causing light attenuation. Interestingly, we documented a high productivity role of the bare sediments compared to the vegetated-canopies of the soft-sediment sites (*i*.*e*., SG and MM). The productivity estimates did not confirm the general hypothesis that canopy-forming vegetation are far more productive than soft sediments without macrovegetation [13]. Alternative primary producers represented by a uniform sedimentary coverage of microphytobenthos (approximately a 60% cover of the total average estimated through ArcMap) can contribute to the metabolism of the unvegetated BS site through the benthic microalgal role on system productivity and trophic dynamics *e*.*g*., [11,49,50]. The relatively high community R recorded in all the soft-sediment sites may also indicate an increase in the sediment heterotrophic O_2_ demand through the respiration and bioturbation of the benthic macroinfauna [4,48]. The high respiration rates of the filter-feeder *M*. *edulis*, although seasonally variable due to reproduction periods and food availability, are expected to play a major role on the net heterotrophy of hard bottoms [51,52]. The BM reef shaped a dense heterotrophic community expected to exert an increase in the biological O_2_ demand through respiration compared to other habitats with less abundant macroinvertebrates or with a larger presence of primary producers. We acknowledge that our data on the metabolic budget for the different habitats only represents one sampling season. Thus, we need to generate a more representative data set by means of sampling across different spatial and temporal scales if we want to formally establish meaningful comparisons of oxygen fluxes between habitats, and to build significant relationships between biodiversity metrics and productivity estimates (see section 4.3.).

### Biodiversity and oxygen flux measurements within habitats

Marine benthic habitats are normally systems of intense, but variable metabolism. Thus, the regulation of metabolic processes is related to a combination of environmental drivers, including light, temperature and flow velocity [23,50,53,54]. Understanding the variability of these environmental drivers is key for establishing the role of benthic communities on the ecosystem metabolism of coastal habitats. For instance, the short-term response of the benthic metabolism to the environmental drivers in a given seagrass meadow can be altered by the water flow conditions [55,56]. It is evident from our measurements that the average productivity estimates (*i*.*e*., GPP and R) in the soft-sediment sites were affected by changes in the irradiance and the water flow. For instance, the single water flow direction recorded in the BS site rendered variable productivity estimates over time, thus the highest productivity rates were related to those days with higher flow velocity and PAR conditions. During prolonged AEC deployments (*i*.*e*.,. 3–5 days) we often observed prompt changes in velocity magnitude and direction. Both MM and SG sites showed multiple flow directions with variable velocities and frequencies that affected the productivity estimate. At MM, macrophyte coverage was heterogeneous and patchy, and the highest productivity rates were recorded across the direction sector with the highest PAR conditions and the largest flow velocities (*i*.*e*., Dir B). In the SG site, light conditions were similar and the seagrass was relatively homogeneous across all the sectors, consequently the NEM was similar (~ -3 mmol O_2_ m^-2^ d^-1^) in all three main directions. However, the maximum productivity rates corresponded to the direction sector with the highest flow velocity (*i*.*e*., Dir C).

Light is considered a critical factor controlling the growth and function of benthic producers (*i*.*e*., macrophytes and microphytobenthos), thus governing the ecosystem metabolism response of coastal habitats [48,49,57]. There are a number of physical (*e*.*g*., sediment resuspension and advection, shelf-shading) and water-quality processes (*e*.*g*., nutrient loads and algal proliferation) that can affect light penetration to the seabed at different spatiotemporal scales [8,58]. Water flow has been considered a main driver of photosynthesis rates by modifying the responses of light, nutrient availability and temperature of vegetated beds [55,58]. For instance, moderately reduced flow and turbulence within vegetated beds may indirectly increase light availability by reducing self-shading on macrophyte canopies and by increasing water transparency due to sediment deposition (see [59]). However, vegetated beds can benefit from increased flow and turbulence due to faster removal of undesired substances and intensified exchange between the overlying water column and that within the meadow [55,58].

The hard-bottom sites were the most contrasting habitats in terms of the biodiversity, productivity estimates and water flow measured. The FV site is an exposed community with high annual averaged wave amplitudes and water flow conditions [30]. The unidirectional flow at the FV site was based on only one complete day of data due to sensor damage incurred ~ 35 h into the measurements, and thus no integrated comparisons between days and flow directions are possible. The light availability in the FV site was similar to the autotrophic (BS) and metabolically balanced (MM) soft-sediment sites at the time of the study. However, it has been suggested that a high proportion of the *F*. *vesiculosus* biomass is photosynthetically-active when compared to other vegetation species such as seagrass, and thus are more efficient at photosynthesizing [60]. The large and photosynthetically-active canopy standing biomass from the FV site is present year-round (see [30]), turning this site into a net autotrophic system for most of the year, with higher annual NEM values than some of the most productive seagrass beds worldwide [30]. The O_2_ fluxes recorded in the BM reef represent only respiration that can be quantified within the different direction sectors. The blue mussel cover was homogenous and abundant in all the sectors, and the multiple flow velocity and frequency was variable, and thus not directly related to the metabolic rates. However, physical episodic drivers such as upwelling occurrences or high flow velocity-induced O_2_ uptakes due to *e*.*g*., advective flushing of anoxic pore-waters and resuspension might have a major role on the variability of the eddy fluxes measured in the BM reef.

### Promising leads and ways forward: A future perspective

An improved understanding of seafloor metabolism requires an appropriate characterization of the community biodiversity. The relationship between benthic community biomass and seafloor metabolism measured by AEC (*i*.*e*., GPP and R) provides a compelling approach for meeting this requirement. The biomass of marine benthic organisms can be easily measured, enabling direct comparison across benthic habitats regardless of the specific structural biodiversity components. Importantly, when trying to establish links between biodiversity metrics and AEC flux measurements, it is key to have a broad ecological knowledge of the system under study, *e*.*g*., through identification of the prevailing structural features and the phenology of the dominant organism that may dictate the productivity rates of the system.

We acknowledge that the biodiversity metrics and the AEC flux measurements in this study were only taken once per habitat. The high natural variability of most of the benthic ecosystem processes, and the potential importance of scaling issues, imply that the relationship between biodiversity metrics and productivity rates in the seafloor will change at different spatiotemporal scales [1]. Thus, the coastal habitats from the Baltic Sea exhibit highly heterogeneous seafloors bound to constant changes in the environmental drivers and in the seafloor features. Regular changes in the environmental conditions such as current velocity, temperature, nutrients, or light can modify the O_2_ flux dynamics of the seafloor *e*.*g*., [27,55,61]. We regard the present paper as an important step forward, setting the stage for upcoming research focusing on spatial biodiversity-productivity relationships and seasonal across-habitat productivity. Therefore, future research trying to compare and link benthic biodiversity measures with AEC metrics of productivity and respiration needs to increase the replication of the different habitats to cope with increasing spatial heterogeneity. Moreover, it is crucial to conduct seasonal sampling to build confidence in the AEC metabolic measurements and to establish links to biodiversity across different benthic habitats, spanning periods of high productivity in summer to winter conditions with low productivity [24,30]. Also, seasonal sampling is key to detect and understand concurrent changes in biodiversity metrics (*e*.*g*., biomass) and sediment-water O_2_ fluxes. Long-term (2–3 months) observatory platforms (*e*.*g*., landers, sensors, video) for coastal application studies deployed to record physical, biogeochemical and/or biological activity can help to relate major environmental changes in the water (*e*.*g*., algal blooms) with the AEC measurements. Other sampling methods and non-destructive techniques such as photogrammetry, multibeam sonar, or ROV surveys for biodiversity characterization of other specific habitats could be incorporated depending on the habitat-specific requirements [62–64]. The natural heterogeneity of the benthic communities needs to be considered in planning AEC measurements because seafloor heterogeneity increases with scale, affecting the benthic biomass and consequently the NEM of the habitats [3,21,48]. Few empirical studies have documented how the dominant drivers of ecosystem metabolism shift across multiple scales in heterogeneous environments (see [3]). Following our sampling design, coupled relationships between biodiversity metrics (*e*.*g*., biomass) and AEC measurements (*e*.*g*., GPP) could be properly established by increasing the precision of sampling (*i*.*e*., the number of replicates), consequently improving the estimates of variation (*i*.*e*., statistical power), and the confidence of the conclusions drawn from potential biological links. Analysing the strength and variability of such coupled biological relationships following steep environmental and biodiversity gradients within habitats can provide useful ecological insight on the role of biodiversity for ecosystem functioning and benthic community health [3,14,65]. For temporal studies on the same habitat (*i*.*e*., revisiting sites), we recommend not to oversample to avoid destruction of the habitat biodiversity contributors that could potentially affect the outcome of the productivity rates. Given the novelty of the AEC measurements in the aquatic environment, there is limited experience with the approach and several aspects of the technique still need to be investigated. However, the use of the AEC approach combined with a standardized biodiversity sampling protocol opens new avenues for marine ecology research using various metrics of ecosystem functioning such as light efficiency, standing biomass, macrofauna respiration, nutrient retention or turnover rates as common currencies for potential across-habitat comparisons. Also, the implementation of the AEC technique combining cross-scale interactions provides a new perspective on the role of biodiversity and heterogeneous habitats for ecosystem metabolism, functioning and services at scales of societal relevance and appropriate for coastal management.

## Supporting information

S1 File Habitat biodiversity characterization. Detailed information on the benthic biodiversity (producers and consumers) across all the coastal habitats.
(PDF)

S1 Fig Average percent cover (±SE) of the main biodiversity components. Pictures taken at the five study habitats using quadrats (25 x 25 cm, n = 24 per habitat) placed along the guide-lines (at 1, 3 and 5 m), and estimated by a supervised image classification technique (ArcGIS 10.1, ArcMap). Pictures showed here are random examples of the total number of photographs taken. MPB: microphytobenthos. *F*. *vesiculosus*: *Fucus vesiculosus*.
(TIF)

## References

[1] Snelgrove PVR, Thrush SF, Wall DH, Norkko A. Real world biodiversity-ecosystem functioning: a seafloor perspective. 2014; Trends Ecol Evol. 29:398–405. 10.1016/j.tree.2014.05.002 24932849

[2] Lohrer AM, Thrush SF, Gibbs MM. Bioturbators enhance ecosystem function through complex biogeochemical interactions. 2004; Nature 431:1092–1095. 10.1038/nature03042 15470385

[3] Lohrer AM, Thrush SF, Hewitt JE, Kraan C. The up-scaling of ecosystem functions in a heterogeneous world. 2015; Sci Rep. 5:10349 10.1038/srep10349 25993477 PMC4438619

[4] Glud RN. Oxygen dynamics of marine sediments. 2008; Mar Biol Res. 4:243–289.

[5] Duffy EJ. Why biodiversity is important to the functioning of real-world ecosystems. 2009; Front Ecol Environ. 7(8):437–444.

[6] Mermillod-Blondin F, Françoise-Carcaillet F, Rosenberg R. Biodiversity of benthic invertebrates and organic matter processing in shallow marine sediments: an experimental study. 2005; J Exp Mar Biol Ecol. 315:187–209.

[7] Duarte CM, Middelburg JJ, Caraco N. Major role of marine vegetation on the oceanic carbon cycle. 2005; Biogeosciences. 2(1): 1–8.

[8] Binzer T, Sand-Jensen K, Middelboe A-L. Community photosynthesis of aquatic macrophytes. 2006; Limnol Oceanogr. 51(6): 2722–2733.

[9] Bordeyne F, Migné A, Davoult D. Metabolic activity of intertidal *Fucus* spp. communities: Evidence for high aerial carbon fluxes displaying seasonal variability. 2015; Mar Biol. 162:2119–2129.

[10] MacIntyre HL, Geider RJ, Miller DC. Microphytobenthos: the ecological role of the ‘secret garden’ of unvegetated, shallow-water marine habitats, 1. Distribution, abundance and primary production. 1996; Estuaries. 19:186–201.

[11] Glud RN, Kühl M, Wenzhöfer F, Rysgaard S. Benthic diatoms of a high Arctic fjord (Young Sound, NE Greenland): importance for ecosystem primary production. 2002; Mar Ecol Prog Ser. 238:15–29.

[12] Norkko A, Villnäs A, Norkko J, Valanko S, Pilditch C. Size matters: implications of the loss of large individuals for ecosystem function. 2013; Sci Rep. 3: 2646 10.1038/srep02646 24025973 PMC6505624

[13] Apostolaki ET, Holmer M, Marbà N, Karakassi I. Metabolic Imbalance in Coastal Vegetated (*Posidonia oceanica*) and Unvegetated Benthic Ecosystems. Ecosystems. 2010; 13(10):459–471.

[14] Gammal J, Järnström M, Bernard G, Norkko J, Norkko A. Environmental Context Mediates Biodiversity—Ecosystem Functioning Relationships in Coastal Soft-sediment Habitats. 2018; Ecosystems. Forthcoming 10.1007/s10021-018-0258-9

[15] Berg P, Røy H, Janssen F, Meyer V, Jørgensen BB, Huettel M, de Beer D. Oxygen uptake by aquatic sediments measured with a novel non-invasive eddy-correlation technique. 2003; Mar Ecol Prog Ser. 261:75–83.

[16] Berg P, Røy H, Wiberg PL. Eddy correlation flux measurements: the sediment surface area that contributes to the flux. 2007; Limnol Oceanogr. 52:1672–1684.

[17] Glud RN, Berg P, Hume A, Batty P, Blicher ME, Lennert K, et al Benthic oxygen exchange across hard bottom substrates quantified by eddy correlation in a sub-Arctic fjord. 2010; Mar Ecol Prog Ser. 417:1–12.

[18] Long MH, Berg P, de Beer D, Zieman JC. In situ coral reef oxygen metabolism: an eddy correlation study. 2013; PLoSONE 8:e58581. 10.1371/journal.pone.0058581 PMC3594154 23536798

[19] Hume AC, Berg P, McGlathery KJ. Dissolved oxygen fluxes and ecosystem metabolism in an eelgrass (*Zostera marina*) meadow measured with the eddy correlation technique. 2011; Limnol Oceanogr. 56: 86–96

[20] Reidenbach MA, Berg P, Hume A, Hansen JCR, Whitman ER. Hydrodynamics of intertidal oyster reefs: The influence of boundary layer flow processes on sediment and oxygen exchange. 2013; Limnol Oceanogr: Fluids and environments. 3:225–239.

[21] Rheuban JE, Berg P. The effects of spatial and temporal variability at the sediment surface on aquatic eddy correlation flux measurements. 2013; Limnol Oceanogr Methods. 11:351–359.

[22] Glud RN, Rysgaard S, Turner G, McGinnis DF, Leaky RJG. Biological- and physical- induced oxygen dynamics in melting sea ice of the Fram Strait. 2014; Limnol Oceanogr. 59:1097–1111.

[23] Rheuban JE, Berg P, McGlathery KJ. Multiple timescale processes drive ecosystem metabolism in eelgrass (*Zostera marina*) meadows. 2014a; Mar Ecol Prog Ser. 507:1–13.

[24] Rheuban JE, Berg P, McGlathery KJ. Ecosystem metabolism along a colonization gradient of eelgrass (*Zostera marina* L.) measured by eddy correlation. 2014b; Limnol Oceanogr. 59:1376–1387.

[25] Berg P, Huettel M. Monitoring the seafloor using the non-invasive eddy correlation technique: integrated benthic exchange dynamics. 2008; Oceanography. 21:164–167.

[26] McGinnis DF, Cherednichenko S, Sommer S, Berg P, Rovelli L, et al Simple, robust eddy correlation amplifier for aquatic dissolved oxygen and hydrogen sulfide flux measurements. 2011; Limnol Oceanogr Methods. 9:340–347.

[27] Attard KM, Stahl H, Kamenos NA, Turner G, Burdett HL, Glud RN. Benthic oxygen exchange in a live coralline algal bed and an adjacent sandy habitat: an eddy covariance study. 2015; Mar Ecol Prog Ser. 535:99–115.

[28] Berg P, Long MH, Huettel M, Rheuban JE, McGlathery KJ, et al Eddy correlation measurements of oxygen fluxes in permeable sediments exposed to varying current flow and light. 2013; Limnol Oceanogr. 58:1329–1343.

[29] McGinnis DF, Sommer S, Lorke A, Glud RN, Linke P. Quantifying tidally-driven benthic oxygen exchange across permeable sediments: an aquatic eddy correlation study. 2014; J Geophys Res. 119:6918–6932.

[30] Attard KM, Rodil IF, Berg P, Norkko J, Norkko A, Glud R. Seasonal metabolism and carbon export potential of a key coastal habitat: the perennial canopy-forming macroalga *Fucus vesiculosus*. 2018; Limnol Oceanogr. 10.1002/lno.11026

[31] Attard KM, Glud RN, McGinnis DF, Rysgaard S. Seasonal rates of benthic primary production in a Greenland fjord measured by aquatic eddy-correlation. 2014; Limnol Oceanogr. 59:1555–1569.

[32] Rovelli L, Attard KM, Binley A, Heppell CM, Stahl H, Trimmer M, et al Reach-scale river metabolism across contrasting sub-catchment geologies: Effect of light and hydrology. 2017; Limnol Oceanogr. 62:381–399. 10.1002/lno.10619 PMC5724700 29242670

[33] Rovelli L, Attard KM, Bryant LD, Flögel S, Stahl H, Roberts JM, et al Benthic O_2_ uptake of two cold-water coral communities estimated with the non-invasive eddy correlation technique. 2015; Mar Ecol Prog Ser. 525:97–104.

[34] Long MH, Berg P, McGlathery KJ, Zieman JC. Sub-tropical seagrass ecosystem metabolism measured by eddy covariance. 2015; Mar Ecol Prog Ser. 529:75–90.

[35] Kautsky H. Quantitative distribution of plant and animal communities of the phytobenthic zone in the Baltic Sea. Contributions from the Askö Laboratory, University of Stockholm, Sweden. 1989; 35:1–80.

[36] Westerbom M, Mustonen O, Kilpi M. Distribution of a marginal population of *Mytilus edulis*: responses to biotic and abiotic processes at different spatial scales. 2008; Mar Biol. 153:1153–1164.

[37] Rumohr H, Brey T, Ankar S. A compilation of biometric conversion factors for benthic invertebrates of the Baltic Sea. Baltic Marine Biologists 1987; Publication No. 9. 56 pp.

[38] Brey T. Population dynamics in benthic invertebrates. A virtual handbook. 2001; Alfred Wegener Institute for Polar and Marine Research, Germany http://www.awi-bremerhaven.de/Benthic/Ecosystem/FoodWeb/Handbook/main.html.

[39] Anderson MJ. Gorley RN. Clarke KR. PERMANOVA + for FRIMER: guide to software and statistical methods. 2008; Plymouth, UK: PRIMER-E.

[40] Clarke KR. Non‐parametric multivariate analyses of changes in community structure. 1993; Aust J Ecol. 18:117–143.

[41] Clarke KR. Gorley RN. PRIMER v6: User Manual/Tutorial. 2006; PRIMER-E, Plymouth, 192 pp.

[42] Thrush SF, Gray JS, Hewitt JE, Ugland KI. Predicting the effects of habitat homogenization on marine biodiversity. 2006; Ecol Appl. 16(5):1636–1642. 17069359 10.1890/1051-0761(2006)016[1636:pteohh]2.0.co;2

[43] Kiirikki M, Lehvo A. Life strategies of filamentous algae in the northern Baltic Proper. 1997; Sarsia 82:259–267.

[44] Norkko A, Bonsdorff E. Rapid zoobenthic community responses to accumulations of drifting algae. 1996; Mar Ecol Prog Ser. 131:143–157.

[45] Tait LW, Schiel DR. Dynamics of productivity in naturally structured macroalgal assemblages: Importance of canopy structure on light-use efficiency. 2011; Mar Ecol Prog Ser. 421:97–107. 10.3354/meps08909

[46] Migné A, Golléty C, Davoult D. Effect of canopy removal on a rocky shore community metabolism and structure. 2015; Mar Biol. 162:449–457. 10.1007/s00227-014-2592-6

[47] Bordeyne F, Migné A, Davoult D. Variation of fucoid community metabolism during the tidal cycle: Insights from in situ measurements of seasonal carbon fluxes during emersion and immersion. 2017; Limnol Oceanogr. 62(6):2418–2430.

[48] Delgard ML, Deflandre B, Bernard G, Richard M, Kochoni E, Charbonnier C, et al Benthic oxygen exchange over a heterogeneous *Zostera noltei* meadow in a temperate coastal ecosystem. 2016; Mar Ecol Prog Ser. 543:55–71.

[49] MacIntyre HL, Gider RJ, Miller DC. Microphytobenthos: The ecological role of the “secret garden” of unvegetated, shallow-water marine habitats. I. Distribution, abundance and primary production. 1996; Estuaries. 19(2):186–201.

[50] Longphuirt SN, Clavier J, Grall J, Chauvaud L, Le Loc´h F, Le Berre I, et al Primary production and spatial distribution of subtidal microphytobenthos in a temperate coastal system, the Bay of Brest, France. 2007; Estuar Coast Shelf Sci. 74:367–380.

[51] Tagliarolo M, Clavier J, Chauvaud L, Koken M, Grall J. Metabolism in blue mussel: intertidal and subtidal beds compared. 2012; Mar Ecol Prog Ser. 17:167–180.

[52] Landes A, Dolmer P, Poulsen LK, Jens Petersen JK, Vismann B. Growth and Respiration in Blue Mussels (*Mytilus* spp.) from Different Salinity Regimes. 2015; J Shellfish Res. 34(2):373–382.

[53] Binzer T, Borum J, Pedersen O. Flow velocity affects internal oxygen conditions in the seagrass *Cymodocea nodosa*. 2005; Aquat Bot. 83:239–247.

[54] Ralph PJ, Durako MJ, Enríquez S, Collier CJ, Doblin MA. Impact of light limitation on seagrasses. 2007; J Exp Mar Biol Ecol. 350:176–193.

[55] Peralta G, Brun FG, Pérez-Lloréns JL, Bouma TJ. Direct effects of current velocity on the growth, morphometry and architecture of seagrasses: a case study on *Zostera noltii*. 2006; Mar Ecol Prog Ser. 327:135–142.

[56] Koopmans D, Holtappels M, Chennu A, Weber M, de Beer D. The response of seagrass (*Posidonia oceanica*) meadow metabolism to CO_2_ levels and hydrodynamic exchange determined with aquatic eddy covariance. in review 2018; Biogeosci Discuss. 10.5194/bg-2018-199.

[57] Lee K-S, Park SR, Kim YK. Effects of irradiance, temperature, and nutrients on growth dynamics of seagrasses: A review. 2007; J Exp Mar Biol Ecol. 350:144–175.

[58] Koch EW, Gust G. Water flow in tide-and wave-dominated beds of the seagrass *Thalassia testudinum*. 1999; Mar Ecol Prog Ser. 184:63–72.

[59] Koch EW. Beyond light: physical, geological, and geochemical parameters as possible submersed aquatic vegetation habitat requirements. 2001; Estuaries. 24:1–17.

[60] Markager S, Sand-Jensen K. Light requirements and depth zonation of marine macroalgae. 1992; Mar Ecol Prog Ser. 88:83–92.

[61] Ouisse V, Migné A, Davoult D. Seasonal variations of community production, respiration and biomass of different primary producers in an intertidal *Zostera noltii* bed (western English Channel, France). 2010; Hydrobiologia. 649:3–11.

[62] Micallef A, Le Bas TP, Huvenne VAI, Blondel P, Huhnerbach V, Deidun A. A multi-method approach for benthic habitat mapping of shallow coastal areas with high-resolution multibeam data. 2012; Cont Shelf Res. 39–40:14–26.

[63] Robert K, Hevenne VAI, Georgiopoulou A, Jones DOB, Marsh L, Carter GDO, et al New approaches to high-resolution mapping of marine vertical structures. 2017; Sci Rep. 7: 9005 | 10.1038/s41598-017-09382-z 28827612 PMC5567197

[64] Thorngren L, Holthuis TD, Lindegarth S, Lindegarth M. Developing methods for assessing abundance and distribution of European oysters (*Ostrea edulis*) using towed video. 2017; Plos One 12 (11): e0187870 10.1371/journal.pone.0187870 29141028 PMC5687756

[65] Villnäs A, Norkko A. Benthic diversity gradients and shifting baselines: implications for assessing environmental status. 2011; Ecol Appl. 21(6):2172–2186. 21939052 10.1890/10-1473.1

