# Supplementary material for: Towards a sampling design for characterizing habitat-specific benthic biodiversity related to oxygen flux dynamics using Aquatic Eddy Covariance
Source: PLoS One. 2019 Feb 4;14(2):e0211673. doi: 10.1371/journal.pone.0211673 (PMC6361453; doi:10.1371/journal.pone.0211673)
Supplement: S1 File — Detailed information on the benthic biodiversity (producers and consumers) across all the coastal habitats. (PDF) [file pone.0211673.s001.pdf]

Towards a sampling design for characterizing habitat-specific benthic biodiversity related to oxygen flux dynamics using Aquatic Eddy Covariance

Supporting information

Rodil IF[1,2], Attard KM[1,3], Norkko J[1], Glud RN[3], Norkko A[1,2]

[1]Tvärminne Zoological Station, University of Helsinki, Hanko, Finland

[2]Baltic Sea Centre, Stockholm University, Stockholm, Sweden

[3]University of Southern Denmark, Odense, Denmark

Rodil I.F., Tvärminne Zoological Station, Hanko, Finland, ivan.rodil@helsinki.fi

Attard K.M., Tvärminne Zoological Station, Hanko, Finland, karl.attard@biology.sdu.dk

Norkko J., Tvärminne Zoological Station, Hanko, Finland, joanna.norkko@helsinki.fi

Glud R.N., University of Southern Denmark, Odense, Denmark, rnglud@biology.sdu.dk

Norkko A., Tvärminne Zoological Station, Hanko, Finland, alf.norkko@helsinki.fi

S1 File. Benthic community analysis and habitat biodiversity characterization. Detailed information on the benthic biodiversity (producers and consumers) across all the coastal habitats.

We divided all the macroinvertebrate species into two different groups to compare the benthic assemblages from soft and hard substrates: (1) those species associated to the macrovegetation (i.e. epifauna), and (2) those species either attached directly to the rock or buried in the sediment (i.e. seabed fauna). We used a canonical analysis of principal coordinates (CAP), based on Bray-Curtis matrices of abundance data, to find axes that best discriminate habitat-specific assemblages in the multivariate space [39]. Then, we identified which species characterised better the differences between the benthic assemblages overlaying Spearman rank (> 0.3) correlation vectors of species abundances with the two main canonical axes. The nature of the benthic groupings identified with the constrained (CAP) ordination was further explored by applying the similarity percentages analysis (SIMPER) to determine the contribution of individual species to the average similarity in the habitat-specific assemblages [40].

The CAP (Fig. S1) in combination with PERMANOVA (Pseudo-$F_{7,56}$ = 19.9; $p < 0.001$) identified habitat-specific assemblages. Our results showed strong correlations ($p < 0.001$; δ_1 = 0.97, δ_2 = 0.90) between the abundance data cloud and the habitat-specific benthic groups (Fig. S2). The hard-bottom assemblages (i.e. FV and BM) and both the SG and MM epifauna assemblages, cluster together, demonstrating a similar abundance and composition (Fig. S1). The macroinfauna assemblages from the soft-sediment habitats (i.e. SGbed, MMbed and BS) also cluster together showing high similarities among them (Fig. S1). PERMANOVA analysis (Pseudo-$F_{7,56}$ = 19.9; $p < 0.001$) identified habitat-specific assemblages and similarities (Table S1). Crustaceans were absent from the BS and MM macroinfauna assemblages (i.e. samples from the sediment core), and no polychaetes were collected from the epifauna assemblages of the vegetated sites (Fig. S2-S3). The largest contribution (Table S2-S3) to the macroinfauna corresponded to the bivalves *Macoma balthica* and *Cerastoderma glaucum*, the gastropod *Peringia ulvae* and the polychaetes *Hediste*

diversicolor and *Marenzelleria* spp. (Fig. S1). The largest contribution (Table S2-S3) to the epifauna assemblages in the vegetated soft sediments corresponded to the gastropods *P. ulvae* and *Theodoxus fluviatilis,* and the crustaceans *Idotea balthica* and *Amphibalanus improvisus* (Fig. S1). The largest species contribution (Table S2-S3) to the hard bottom communities were *M. edulis* and *T. fluviatilis* (Fig. S1). The crustaceans *Gammarus* spp., and *I. balthica* were found as epifauna in the FV site, whereas *A. improvisus* and *Gammarus* spp. had a high contribution to the BM site (Table S2-S3) (Fig. S1). The total abundance (Pseudo-$F_{7,56}$ = 29.9) was significantly ($p < 0.001$) different across habitats (Fig. S4). The pair-wise tests showed that the habitat with the highest macrofauna abundance ($p < 0.001$) was the BM site, and the habitat with the lowest macrofauna abundance ($p < 0.001$) was the BS site (Fig. S4). Thus, the FV and SG sites showed higher macrofauna abundance than the MM site (i.e. BS < MM < SG = FV < BM; $p < 0.05$). There were significant differences in the density ($F_{1,14}$ = 19.3, SG > MM, t = 4.4; p = 0.01) and length ($F_{1,14}$ = 22.04, SG < MM, t =4.7; p = 0.01) of the macrophytes between MM and SG sites (Fig. S4, Table S4). We recorded a total abundance of 15±1.5 (mean±SE) *F. vesiculosus* m^{-2} at the FV site, with a total average length of 37.3±3.2 cm, and a total biomass of 577±115 g m^{-2} (Fig. S4).

Figure S1. Canonical analyses of principal coordinates (CAP). CAP relates macroinvertebrate species to the benthic compartments (MMep: mixed macrophyte epifauna, MMbed: mixed macrophyte seabed, SGep: seagrass epifauna, SGbed: seagrass seabed, BS: bare sand, FVep: *Fucus* epifauna, FVbed: *Fucus* seabed, BM: Blue mussel bed). Vector overlay of Spearman rank correlations (> 0.30) of individual invertebrate species (full species names as in S1 File) with the CAP axes. Epifauna: macrofauna associated to marine macrophytes. Seabed fauna: soft-sediment macroinfauna or macrofauna attached to the rocky-bottoms.

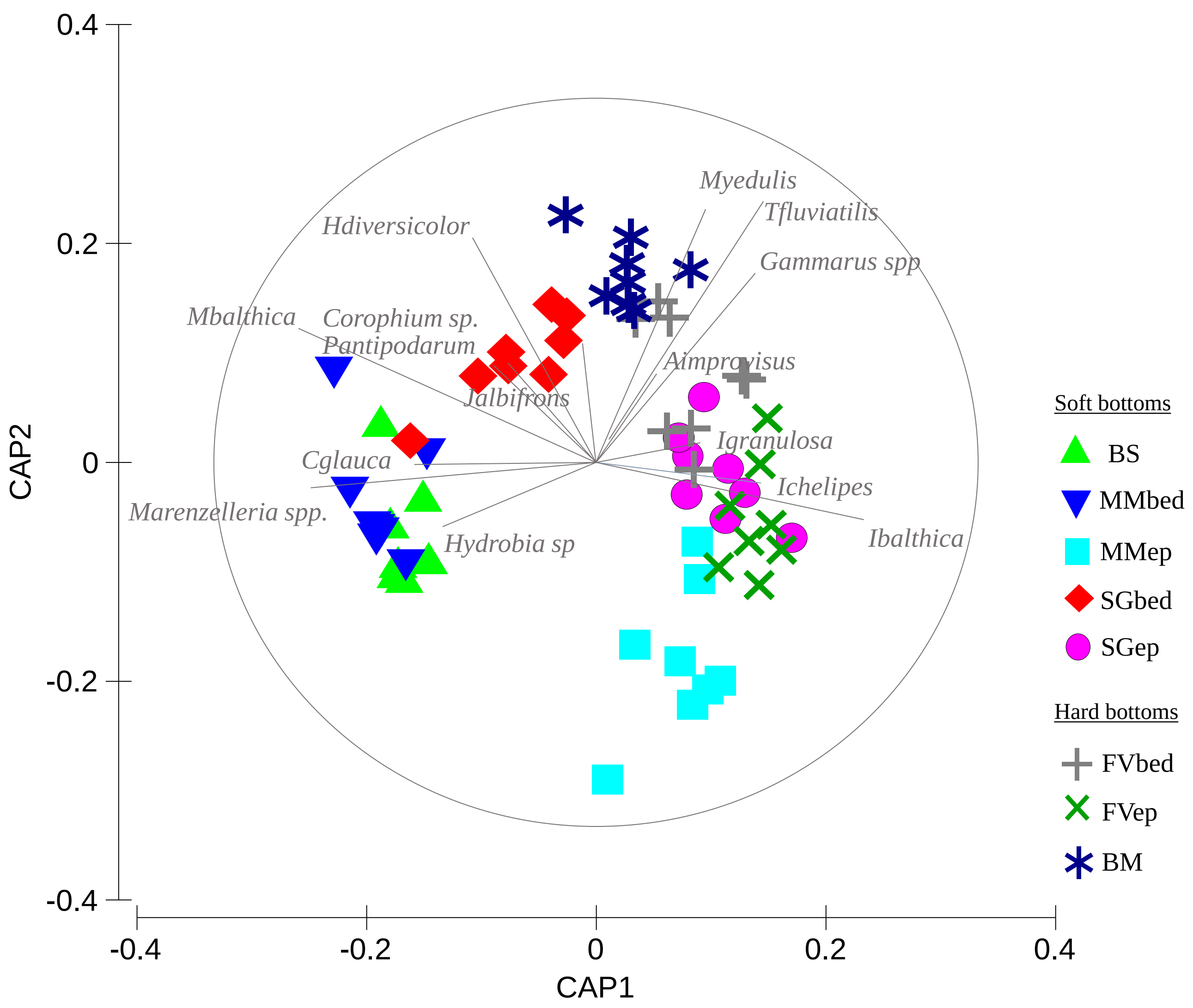

Table S1. Pair-wise tests for macrofauna abundance assemblage comparisons (PERMANOVA, 4999 permutations) between habitat-specific benthic compartments (MMep: mixed macrophyte epifauna, MMbed: mixed macrophyte seabed, SGep: seagrass epifauna, SGbed: seagrass seabed, BS: bare sand, FVep: *Fucus* epifauna, FVbed: *Fucus* seabed fauna, BM: Blue mussel reef) showing t values (all $p < 0.001$ except BS *vs* MMbed) and Bray-Curtis average similarities (%). Epifauna: macrofauna associated to marine macrophytes. Seabed fauna: macroinfauna or macrofauna attached to the rocky-bottoms.

	t values (Average similarity, %)						
Habitat-specific benthic compartments	BS	MMbed	MMepi	SGbed	SGepi	FVbed	FVepi
BS	-	-	-	-	-	-	-
MMbed	1.0 (52.6)	-	-	-	-	-	-
MMepi	3.7 (9.5)	5.5 (11.3)	-	-	-	-	-
SGbed	2.6 (33.0)	3.6 (45.6)	4.6 (21.5)	-	-	-	-
SGepi	4.3 (14.2)	7.4 (16.7)	3.5 (41.7)	5.3 (35.3)	-	-	-
FVbed	3.9 (13.8)	6.2 (15.9)	3.3 (38.2)	3.9 (40.1)	2.4 (60.6)	-	-
FVepi	4.8 (0.0)	7.9 (0.0)	3.3 (39.7)	5.9 (20.3)	2.9 (58.0)	3.8 (42.6)	-
BM	4.7 (12.9)	8.9 (16.2)	5.1 (29.3)	5.7 (40.9)	6.7 (47.5)	3.8 (54.5)	6.4 (36.7)

Table S2. Main species contribution to the similarity (SIMPER, %) of each habitat-specific benthic compartment (i.e. MMep: mixed macrophyte epifauna, MMbed: mixed macrophyte seabed, SGep: seagrass epifauna, SGbed: seagrass seabed, BS: bare sand, FVep: *Fucus* epifauna, FVbed: *Fucus* seabed, BM: Blue mussel reef).

Species	Soft-sediments					Hard-bottoms		
	Bare	MMbed	MMep	SGbed	SGep	FVbed	FVep	BM
Amphibalanus improvisus	-	-	19.4		-	-	-	13.9
Corophium sp.	-	-	-	10.4	-	-	-	-
Gammarus spp.	-	-	11.3	7.6	10.4	7.8	34.7	12.6
Idotea balthica	-	-	26.6	-	29.2	-	29.4	-
Hediste diversicolor	-	-	-	15.2	-	-	-	
Marenzelleria spp.	29.2	27.0	-	9.4	-	-	-	-
Cerastoderma glaucum	-		-	-	-	-	-	-
Macoma balthica	48.2	40.6	-	27.1	6.3	6.1	-	8.6
Mytilus edulis	-	-	21.4	12.4	21.1	41.5	9.2	41.6
Peringia ulvae	17.3	22.8	20.7	10.5	-	6.3	-	-
Potamopyrgus antipodarum	-	-	-	-	-	-	-	-
Theodoxus fluviatilis	-	-	-	-	29.0	29.4	19.2	13.4
Average similarity	45.0	70.1	55.9	67.3	75.7	64.8	67.2	85.0

Table S3. Mean (SE) abundance and biomass of all the macroinvertebrate species collected. Epifauna: macrofauna associated to macrophytes.

Seabed fauna: macroinfauna or macrofauna attached to the rock. Crust: crustacens, Biv: bivalves, Gast: gastropods, Poly: Polychaeta.

			Soft sediments						Hard bottoms			
			Bare sand		Mixed seagrass		Seagrass		Fucus-bed		Blue mussel reef	
Type	Group	Species	Abund	Biom	Abund	Biom	Abund	Biom	Abund	Biom	Abund	Biom
Epifauna	Crust	*Amphibalanus improvisus*	-	-	49(15)	0.2(0.1)	-	-	4(4)	0.01(0.01)	-	-
		Gammarus spp.	-	-	17(5)	0.01(0.01)	70(22)	0.26(0.15)	263(81)	5.88(2.06)	-	-
		Idotea balthica	-	-	53(17)	0.4(0.2)	244(41)	0.88(0.16)	144(48)	0.96(0.21)	-	-
		Idotea chelipes	-	-	-	-	123(55)	0.32(0.15)	43(21)	0.32(0.14)	-	-
		Idotea granulosa	-	-	-	-	-	-	8(5)	0.06(0.04)	-	-
		Jaera albifrons	-	-	-	-	-	-	-	-	-	-
	Biv	*Cerastoderma glaucum*	-	-	-	-	6(6)	0.1(0.1)	-	-	-	-
		Macoma balthica	-	-	-	-	38(17)	0.23(0.12)	-	-	-	-
		Mytilus edulis	-	-	353(207)	0.7(0.5)	120(35)	0.6(0.18)	60(21)	0.32(0.16)	-	-
	Gast	*Peringia ulvae*	-	-	161(77)	0.02(0.01)	42(20)	0.01(0.01)	-	-	-	-
		Radix spp	-	-	5(4)	0.001(0.00)	2(2)	0.002(0.002)	2(2)	0.01(0.01)	-	-
		Theodoxus fluviatilis	-	-	-	-	210(25)	0.39(0.08)	105(46)	0.37(0.19)	-	-
Seabed fauna	Crust	*Amphibalanus improvisus*	-	-	-	-	-	-	13(9)	0.63(0.45)	838(162)	0.31(0.06)
		Corophium spp.	-	-	-	-	891(268)	1.38(0.33)	-	-	-	-
		Gammarus spp.	-	-	-	-	382(127)	3.3(2.5)	59(20)	1.48(0.54)	522(115)	1.53(0.2)
		Idotea balthica	-	-	-	-	255(255)	1.1(1.1)	63(40)		16(7)	0.08(0.05)
		Jaera albifrons	-	-	-	-	-	-	3(3)	0.5(0.41)	13(9)	0.002(0.001)
	Biv	*Cerastoderma glaucum*	127(83)	10.7(8.6)	173(144)	0.9(10.6)	-	-	-	-	9(7)	0.003(0.002)
		Macoma balthica	700(235)	12.7(4.5)	1894(384)	12.7(4.9)	2864(499)	8.63(4.79)	44(18)	0.12(0.05)	409(109)	1.64(0.37)
		Mya arenaria	64(64)	0.1(0.1)	-	-	-	-	-	-	-	-
		Mytilus edulis	-	-	-	-	1782(745)	12.7(4.9)	1916(648)	13.4(4)	46403(3330)	78.8(5.1)
	Poly	*Hediste diversicolor*	64(64)	0.5(0.5)	255(167)	2.3(1.7)	764(167)	3.79(1.39)	19(9)	0.4(0.35)	25(7)	0.52(0.3)
		Marenzelleria spp	509(167)	6.6(2.4)	1337(518)	7.8(3.3)	382(83)	4.42(1.24)	-	-	-	-
	Gast	*Peringia ulvae*	25(8)	0.5(0.5)	1910(584)	0.8(0.3)	258(70)	0.03(0.01)	63(25)	0.03(0.02)	175(84)	0.13(0.1)
		Potamopyrgus antipodarum	-	-	-	-	88(37)	0.04(0.02)	-	-	-	-
		Radix spp	-	-	-	-	-	-	38(19)	0.05(0.03)	-	-
		Theodoxus fluviatilis	-	-	-	-	17(9)	0.77(0.47)	366(69)	1.37(0.28)	575(89)	0.91(0.19)

Figure S2. Mean (+SE) abundance (individuals m^{-2}) and biomass (g dwt m^{-2}) of the main invertebrate groups (Pol: Polychaetes, Biv: Bivalves, Gas: Gastropods and Cru: Crustaceans) collected in the soft sediment habitats.

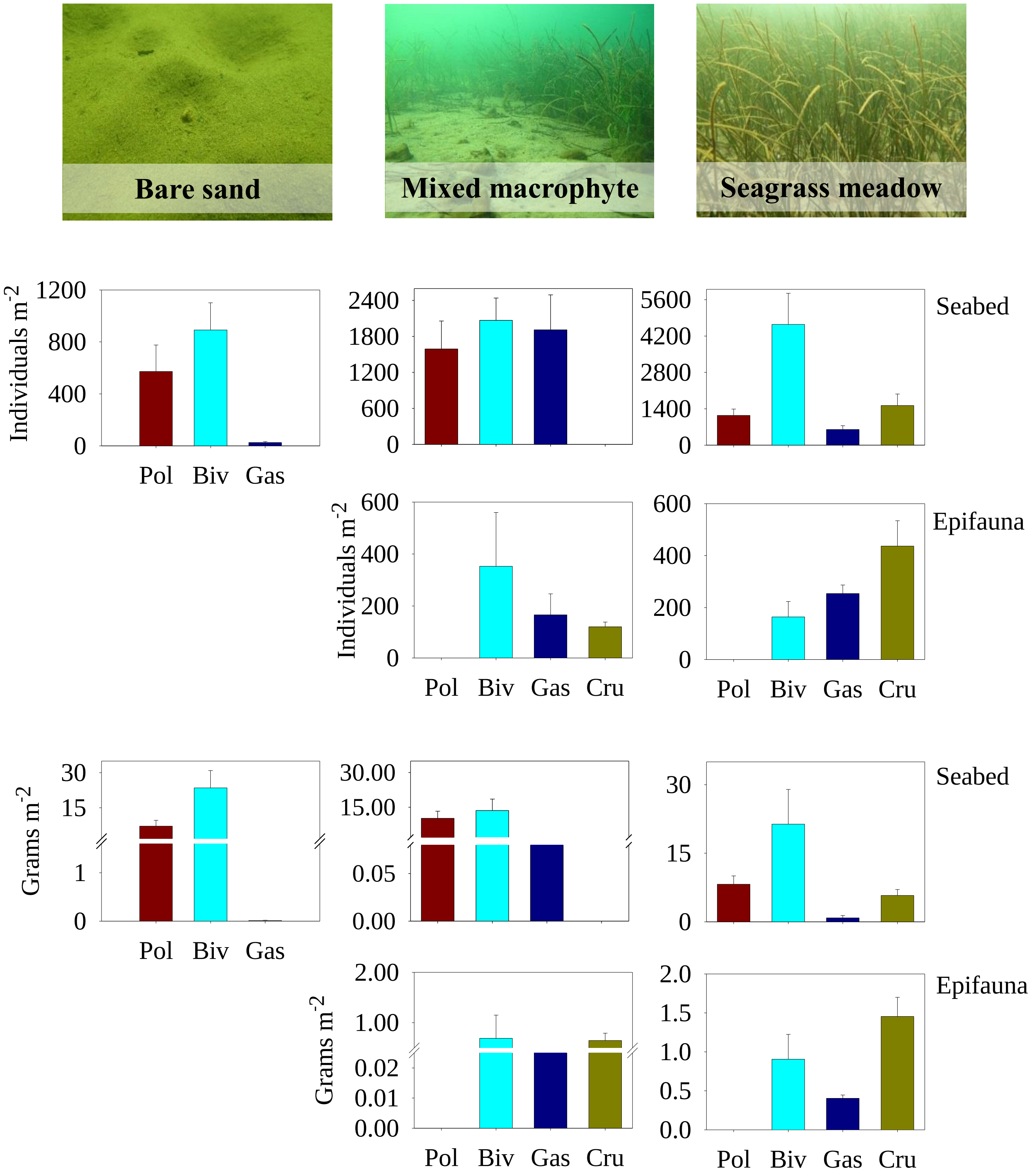

Figure S3. Mean (+SE) abundance (individuals m^{-2}) and biomass (g dwt m^{-2}) of the main invertebrate groups (Pol: Polychaetes, Biv: Bivalves, Gas: Gastropods and Cru: Crustaceans) collected in the hard bottom habitats. *M. edulis data was not included for the BM site (see Fig. 4 for mussel data).

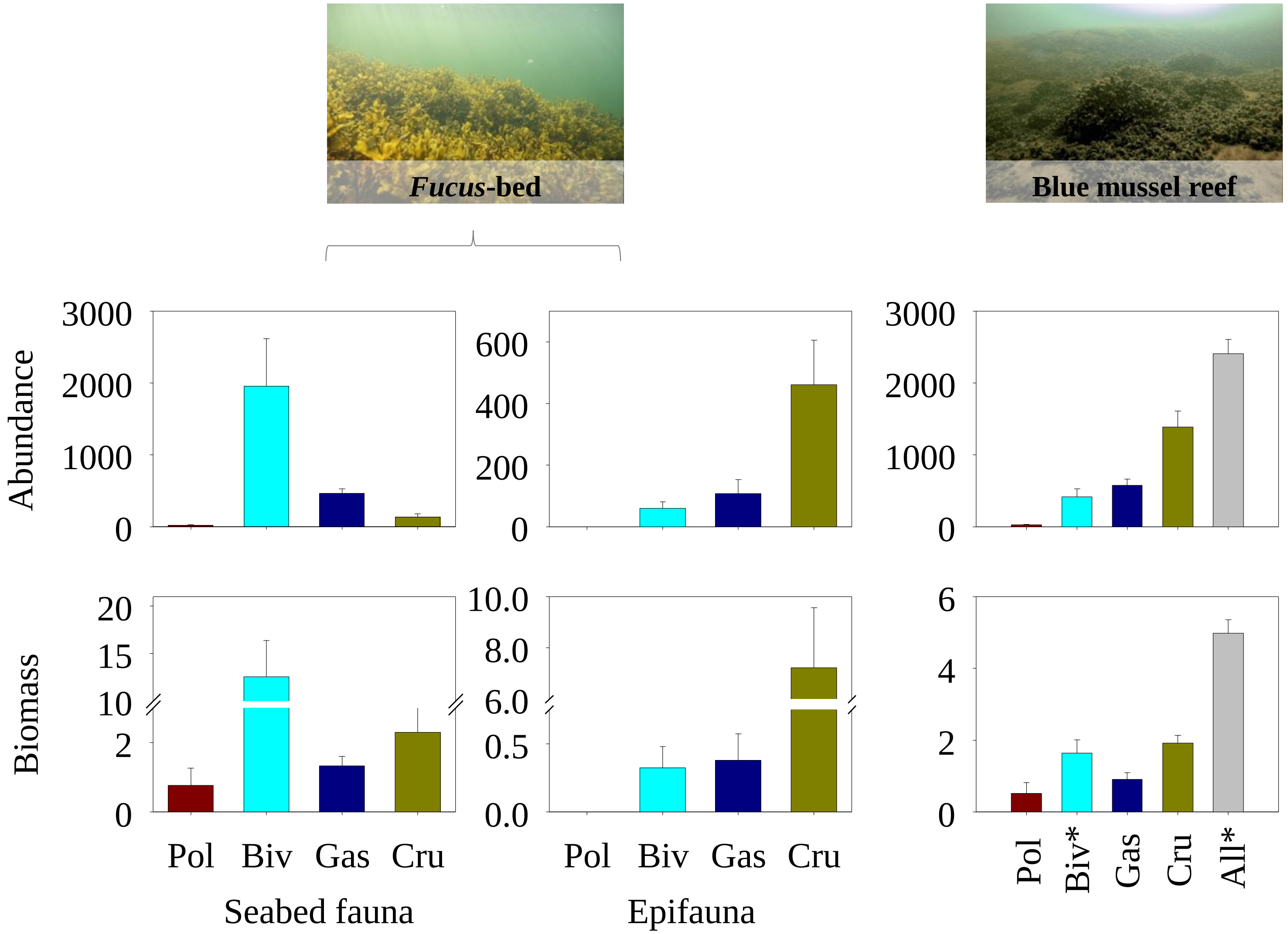

Fig. S4. Mean (+SE) (A) abundance (ind m^{-2}) of the benthic consumers (i.e. total average macrofauna) and (B) the main primary producers from all the study habitats (BS: bare sand, MM: mixed macrophyte, SG: seagrass meadow, FV: bladder-wrack bed made of *F. vesiculosus*, BM: blue mussel reef). Lower case letters: Columns with the same letter indicate that the difference between the means was not statistically significant; columns with different letters indicate that they were significantly different.

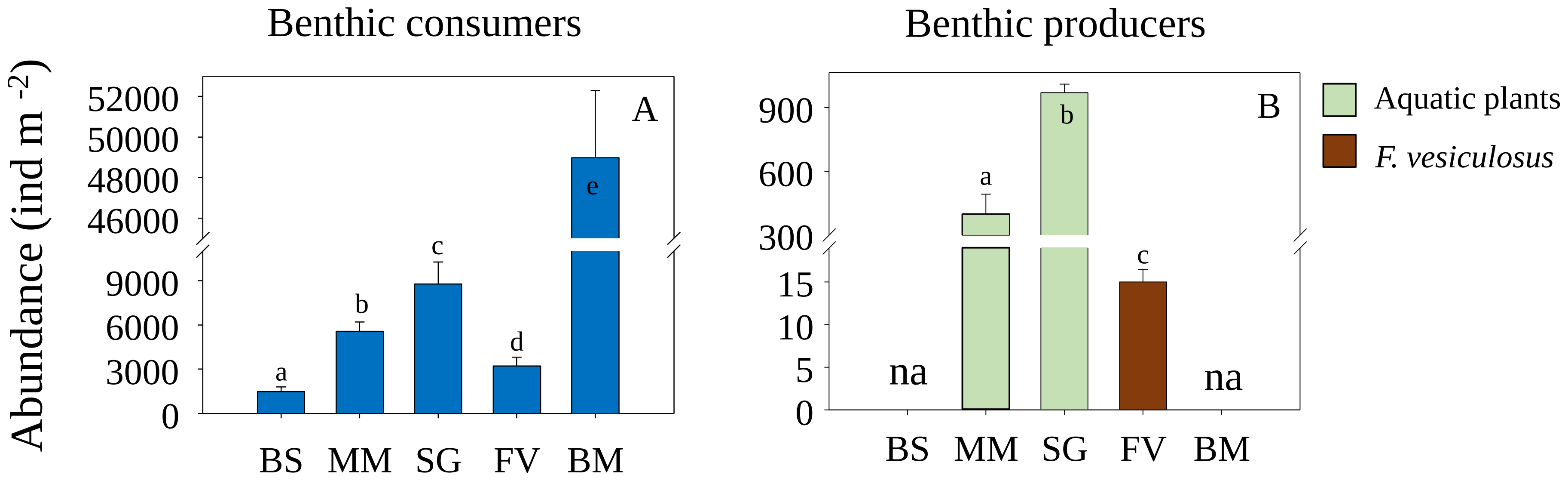

Table S4. Mean (±SE) biodiversity characteristics of the main macrovegetation species. Dry weight (dwt) is shown for above and below ground biomass and total biomass collected at the MM and SG sites.

	Mixed macrophyte (MM)					Seagrass meadow (SG)				
	Density	Biomass (dwt, g m^{-2})			Length	Density	Biomass (dwt, g m^{-2})			Length
Species	(ind m^{-2})	Above	Below	Total	(cm)	(ind m^{-2})	Above	Below	Total	(cm)
Myriophyllum spicatum	28±15	6.8±3.4	0.23±0.14	7±3.5	24.4±8.5	-	-	-	-	-
Potamogeton perfoliatus	2±2	0.04±0.04	0±0	0.04±0.04	1.6±1.6	-	-	-	-	-
Ruppia spp.	66±41.5	4.02±2.8	1.1±0.8	4.1±3.2	6.8±3.4	-	-	-	-	-
Stuckenia pectinata	30±25.7	5.3±5.1	0.1±0.1	5.3±5.1	6±4.2	-	-	-	-	-
Zostera marina	272±92.5	26±9	19.5±8	45.5±16.9	27.2±1.2	950±42.8	56.7±5.6	12.1±1.3	68.8±6.6	25.9±1.2
Zannichellia spp.	-	-	-	-	-	16±16	0.2±0.2	0.03±0.03	0.2±0.2	1.9±1.9
Total	398±94.2	41.1±8.8	20.9±7.8	62±15.9	66±9.5	970±40	56.9±5.5	12.2±1.3	69±6.5	27.8±2
