# Supplementary figures and images for: Towards a sampling design for characterizing habitat-specific benthic biodiversity related to oxygen flux dynamics using Aquatic Eddy Covariance
Source: PLoS One. 2019 Feb 4;14(2):e0211673. doi: 10.1371/journal.pone.0211673 (PMC6361453; doi:10.1371/journal.pone.0211673)

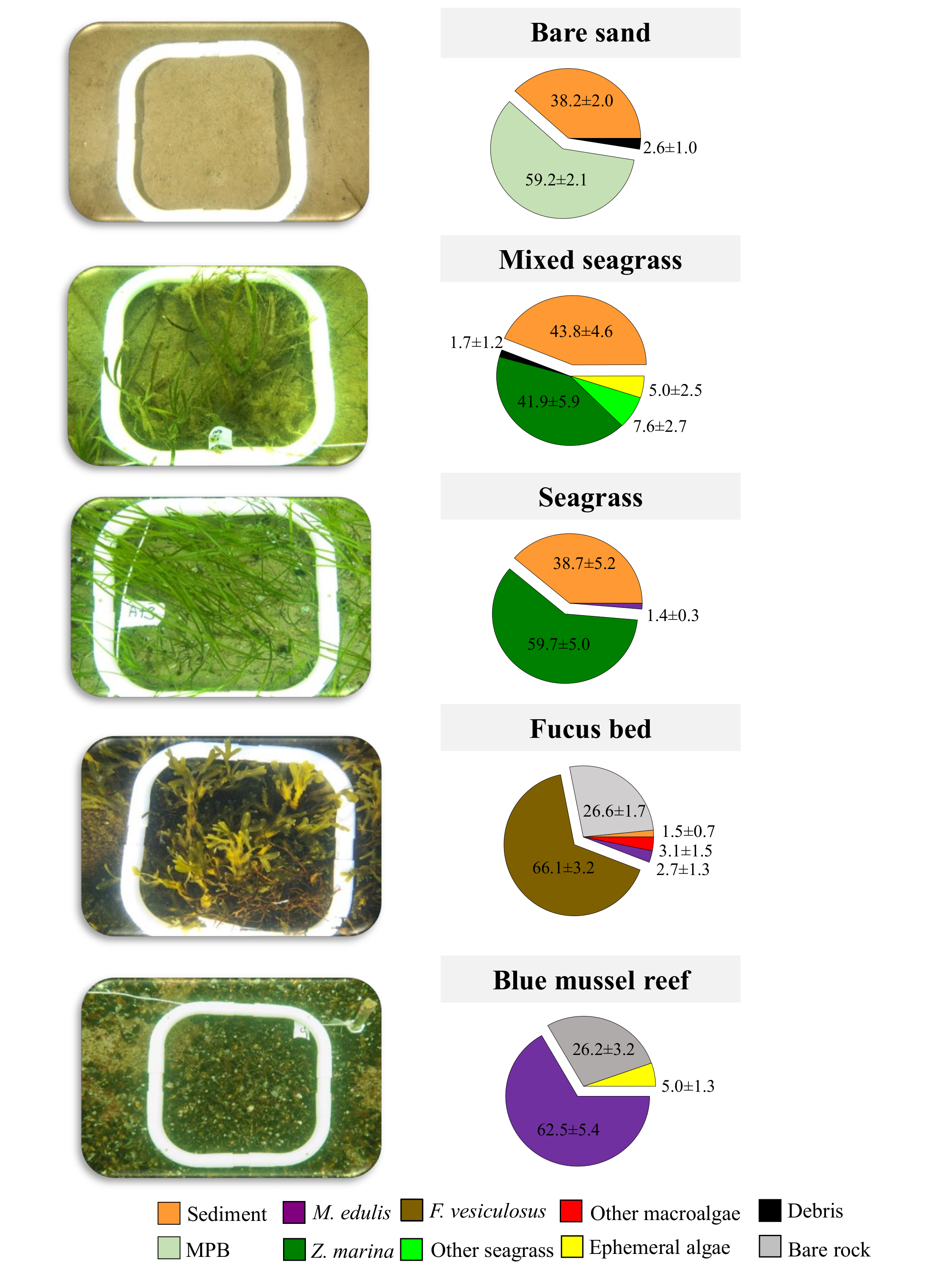

Supplement: S1 Fig — Pictures taken at the five study habitats using quadrats (25 x 25 cm, n = 24 per habitat) placed along the guide-lines (at 1, 3 and 5 m), and estimated by a supervised image classification technique (ArcGIS 10.1, ArcMap). Pictures showed here are random examples of the total number of photographs taken. MPB: microphytobenthos. F. vesiculosus: Fucus vesiculosus. (TIF) [file pone.0211673.s002.tif]
